# Supplementary figures and images for: Pharmacological Gq inhibition induces strong pulmonary vasorelaxation and reverses pulmonary hypertension
Source: EMBO Mol Med. 2024 Jul 8;16(8):7. doi: 10.1038/s44321-024-00096-0 (PMC11319782; doi:10.1038/s44321-024-00096-0)

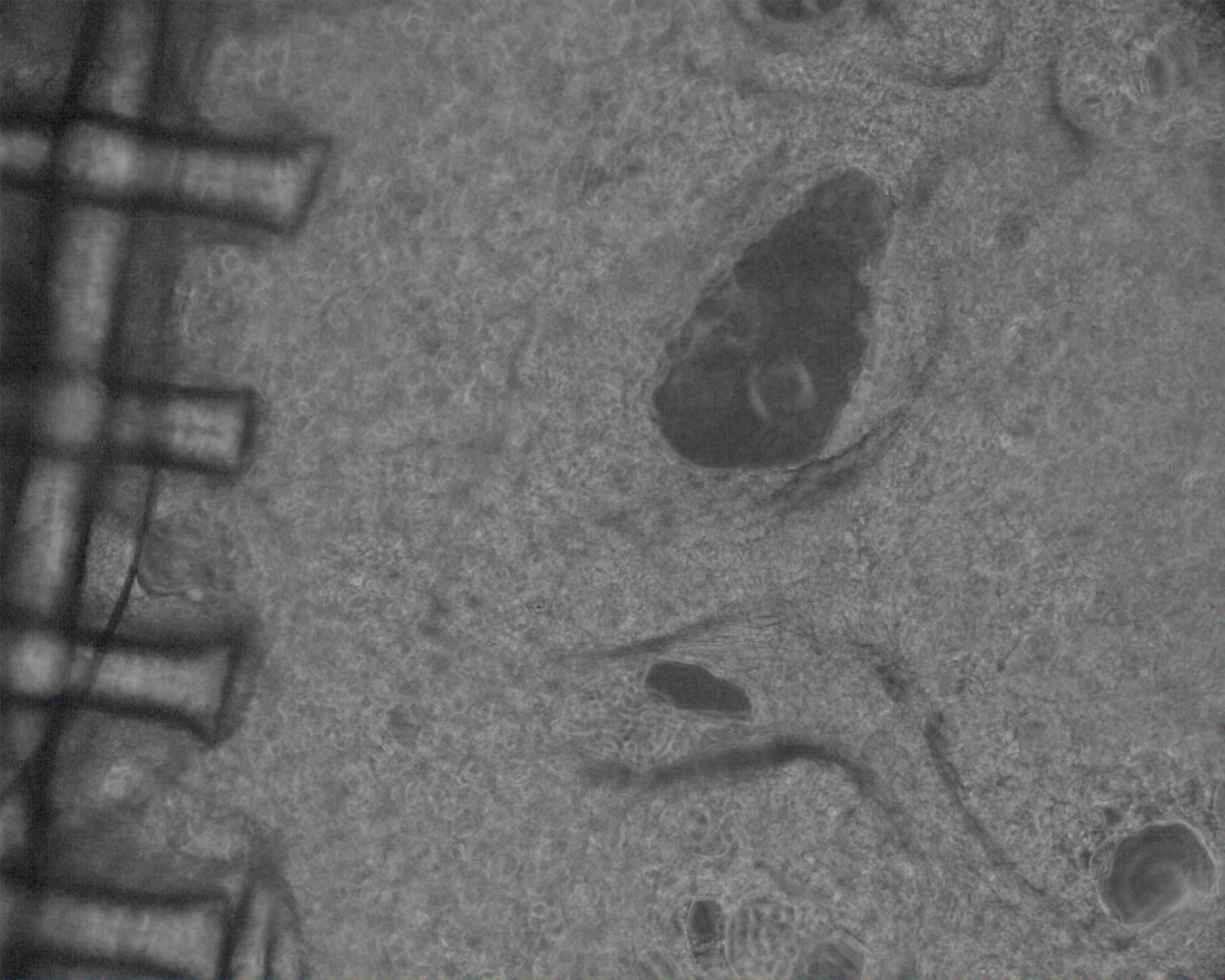

Supplement: Supplementary file 6 — Source data Fig. 5 [file 44321_2024_96_MOESM6_ESM.zip › Figure 5/5A/1 baseline left.bmp]

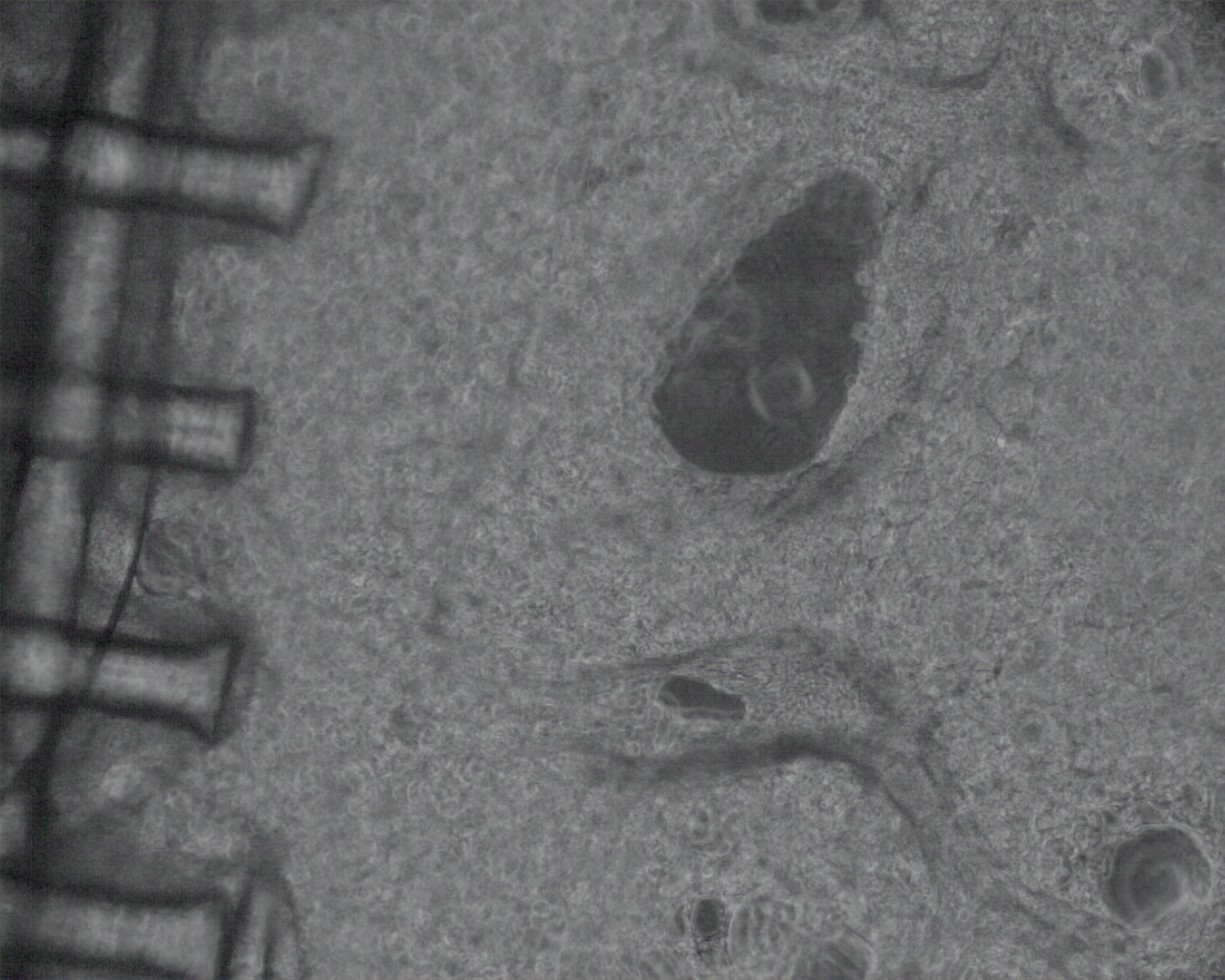

Supplement: Supplementary file 6 — Source data Fig. 5 [file 44321_2024_96_MOESM6_ESM.zip › Figure 5/5A/2 5-HT middle.bmp]

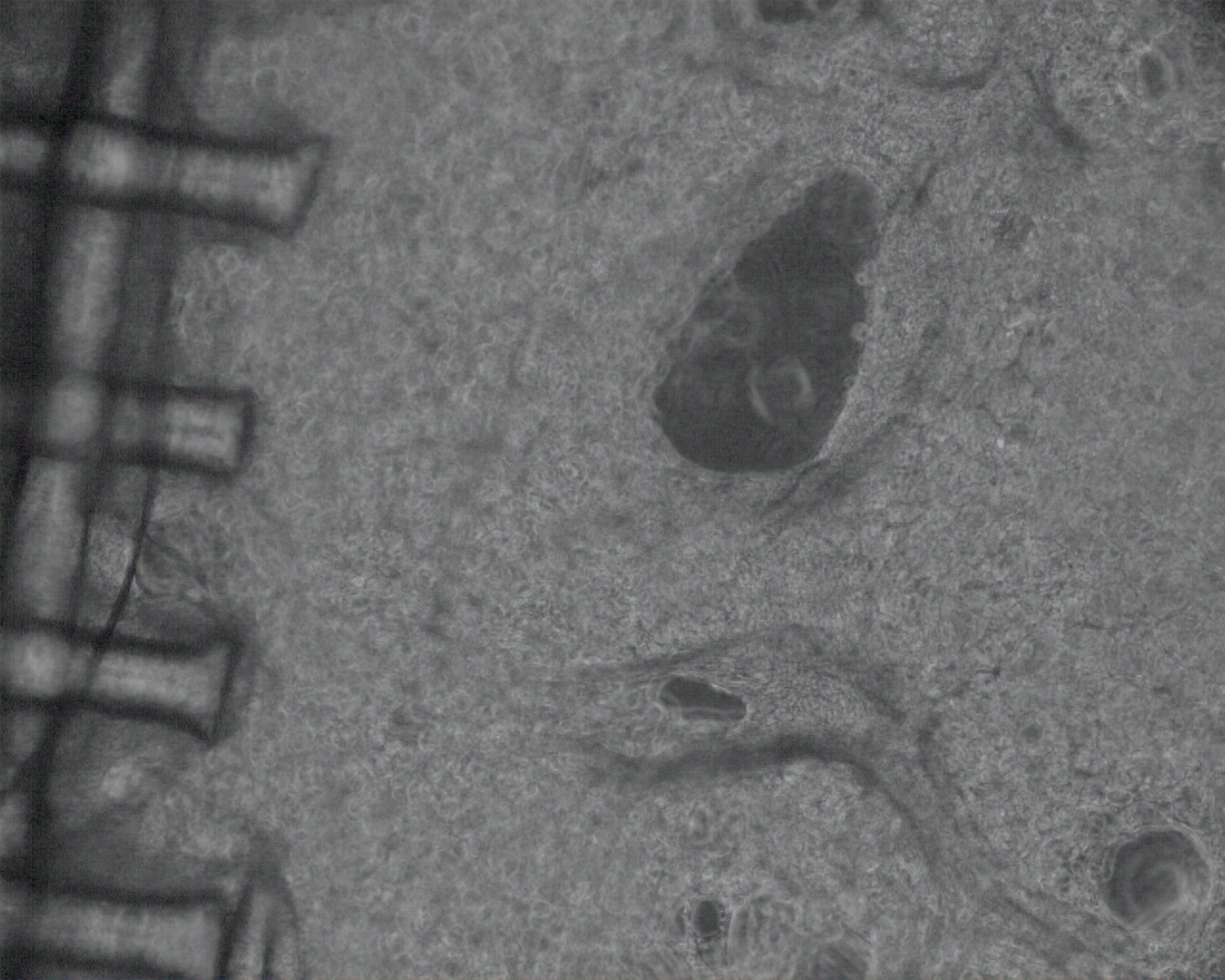

Supplement: Supplementary file 6 — Source data Fig. 5 [file 44321_2024_96_MOESM6_ESM.zip › Figure 5/5A/3 5-HT + DMSO right.bmp]

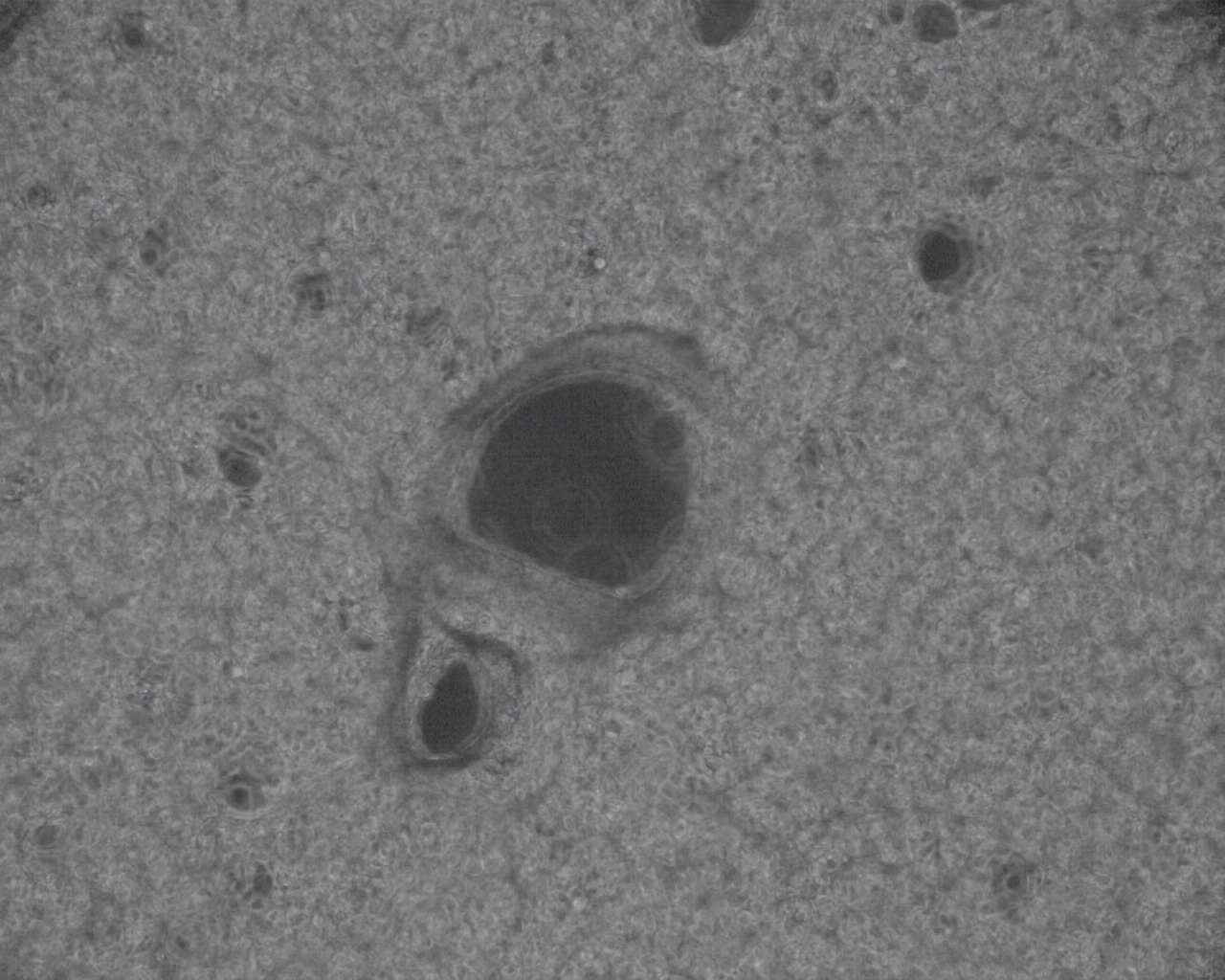

Supplement: Supplementary file 6 — Source data Fig. 5 [file 44321_2024_96_MOESM6_ESM.zip › Figure 5/5B/1 baseline left.bmp]

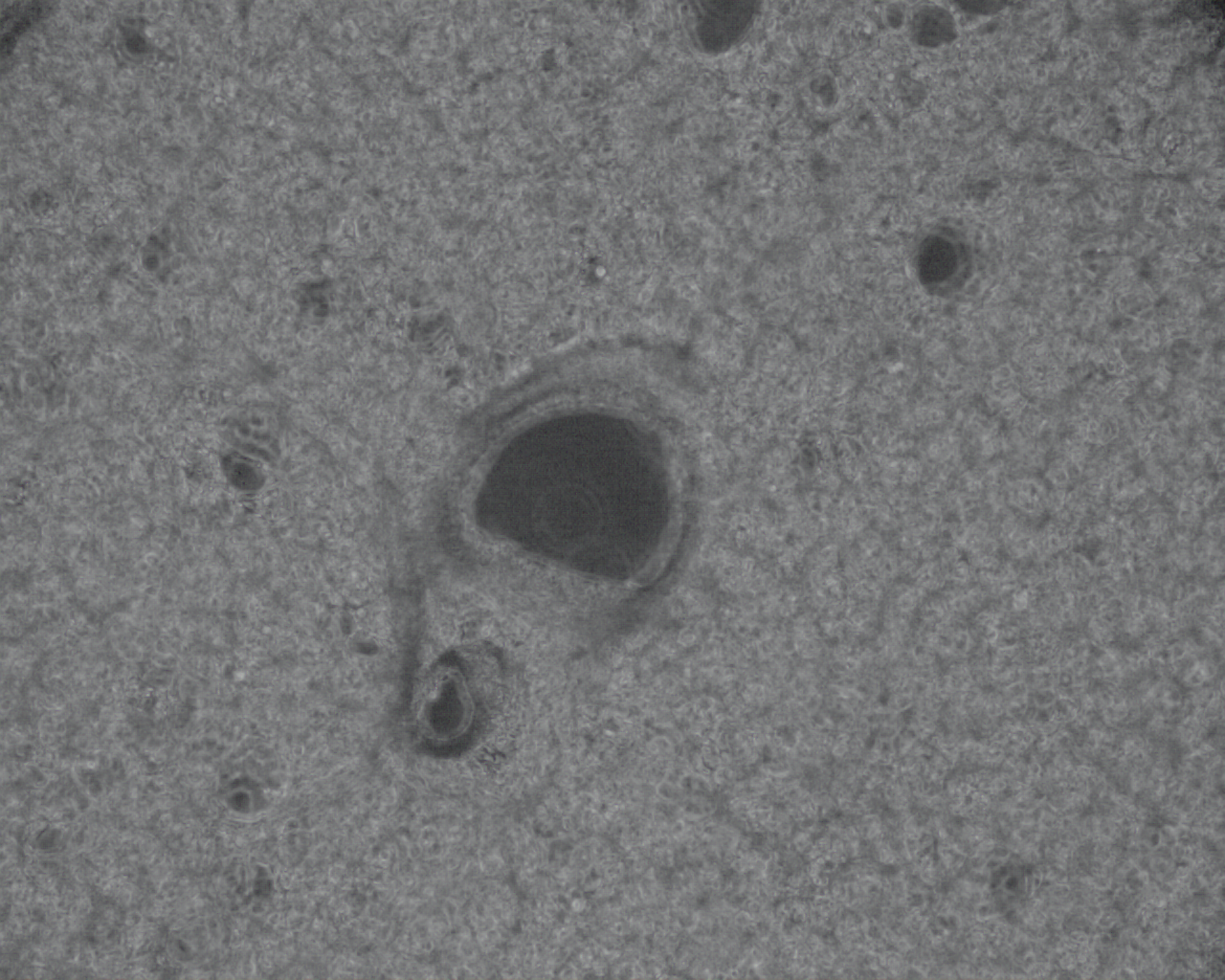

Supplement: Supplementary file 6 — Source data Fig. 5 [file 44321_2024_96_MOESM6_ESM.zip › Figure 5/5B/2 5-HT middle.bmp]

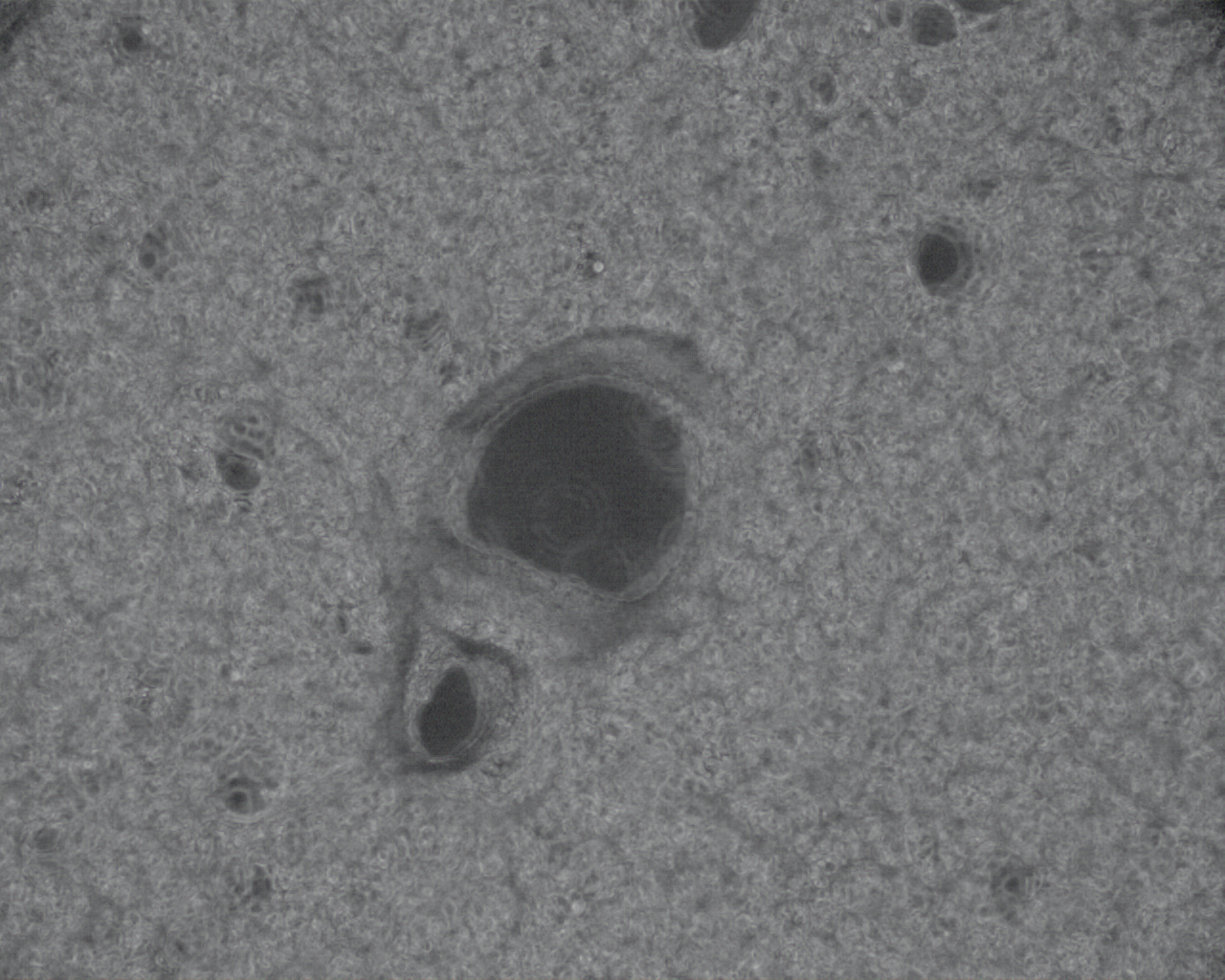

Supplement: Supplementary file 6 — Source data Fig. 5 [file 44321_2024_96_MOESM6_ESM.zip › Figure 5/5B/3 5-HT + FR right.bmp]

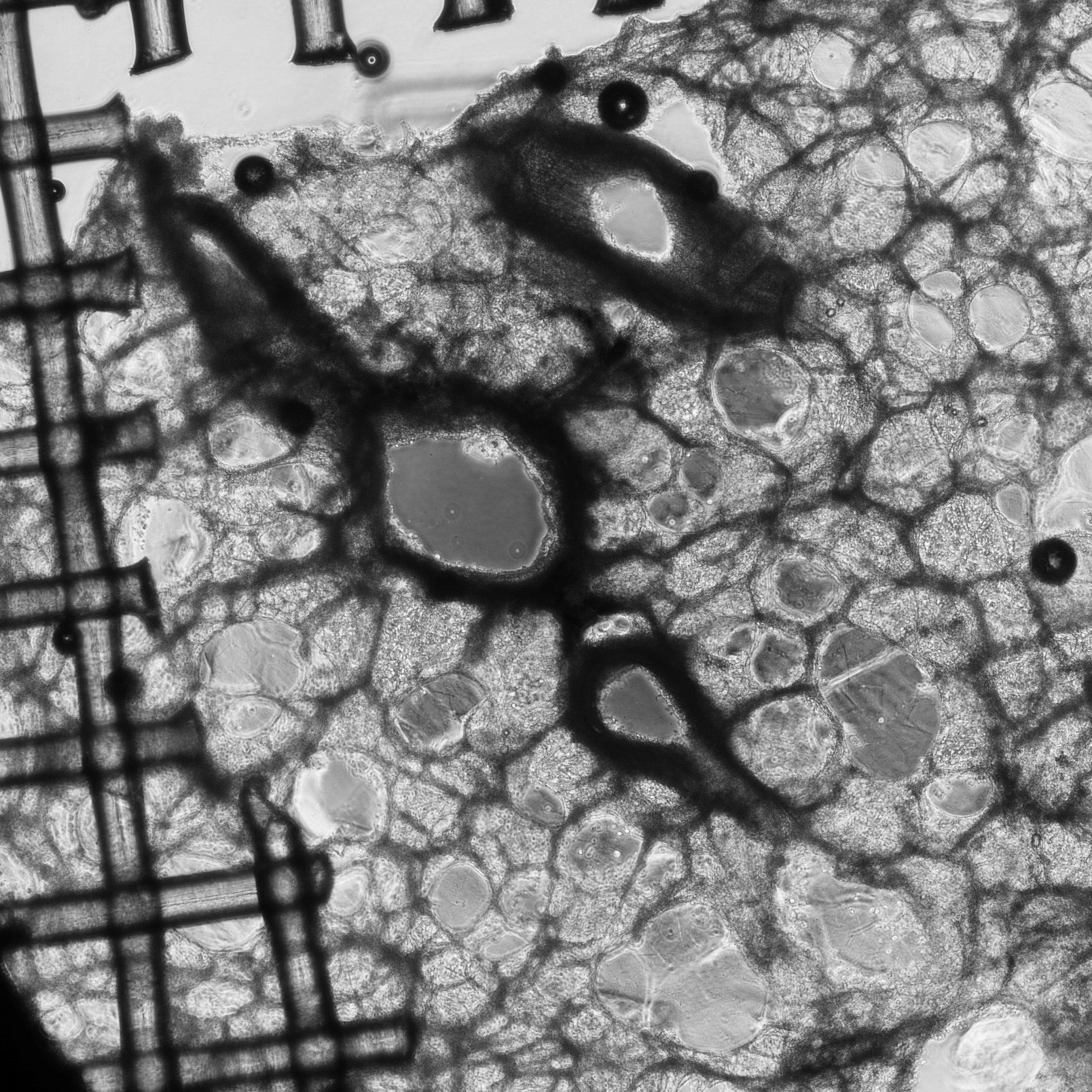

Supplement: Supplementary file 6 — Source data Fig. 5 [file 44321_2024_96_MOESM6_ESM.zip › Figure 5/5D/vessel01-baseline left.tif]

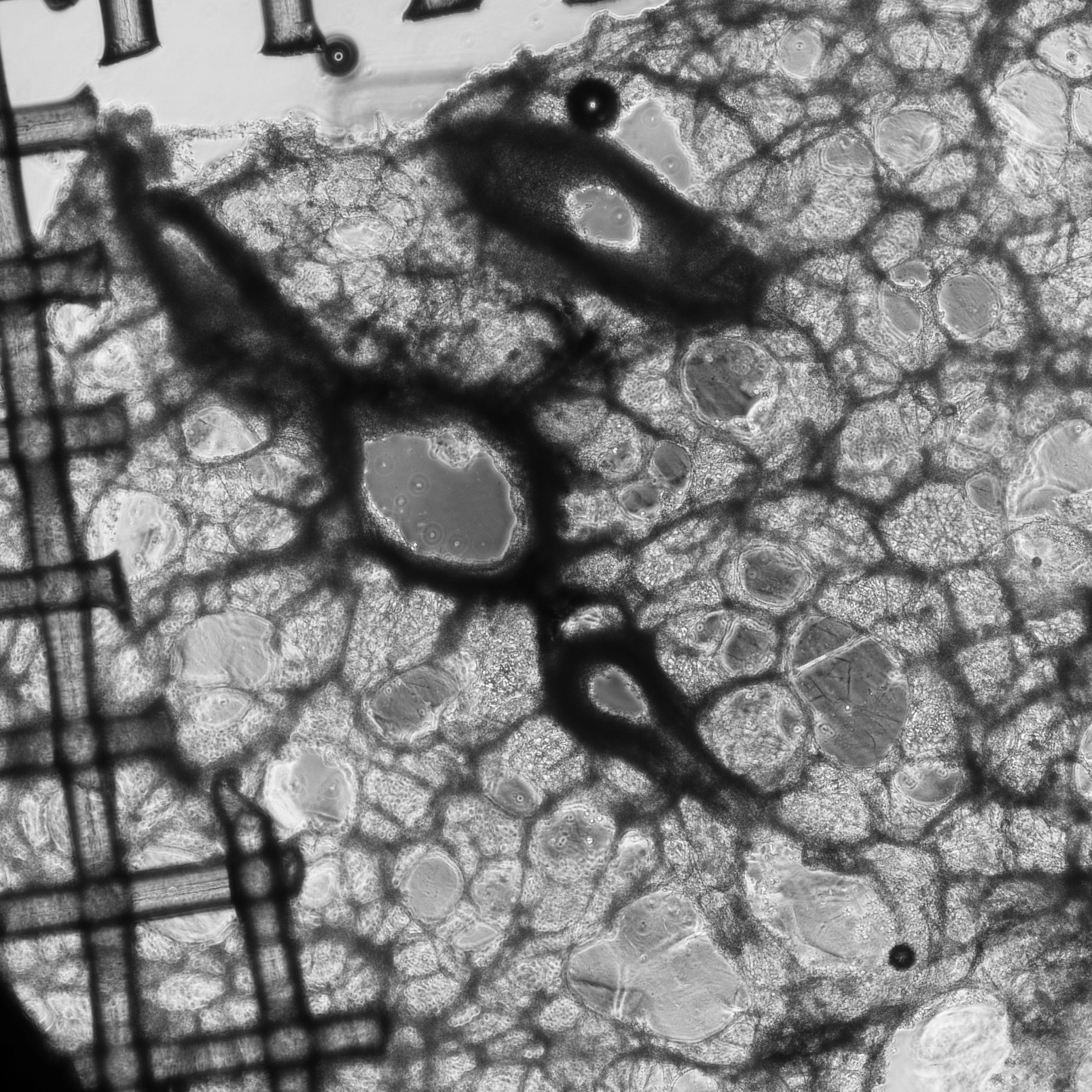

Supplement: Supplementary file 6 — Source data Fig. 5 [file 44321_2024_96_MOESM6_ESM.zip › Figure 5/5D/vessel01-endo middle.tif]

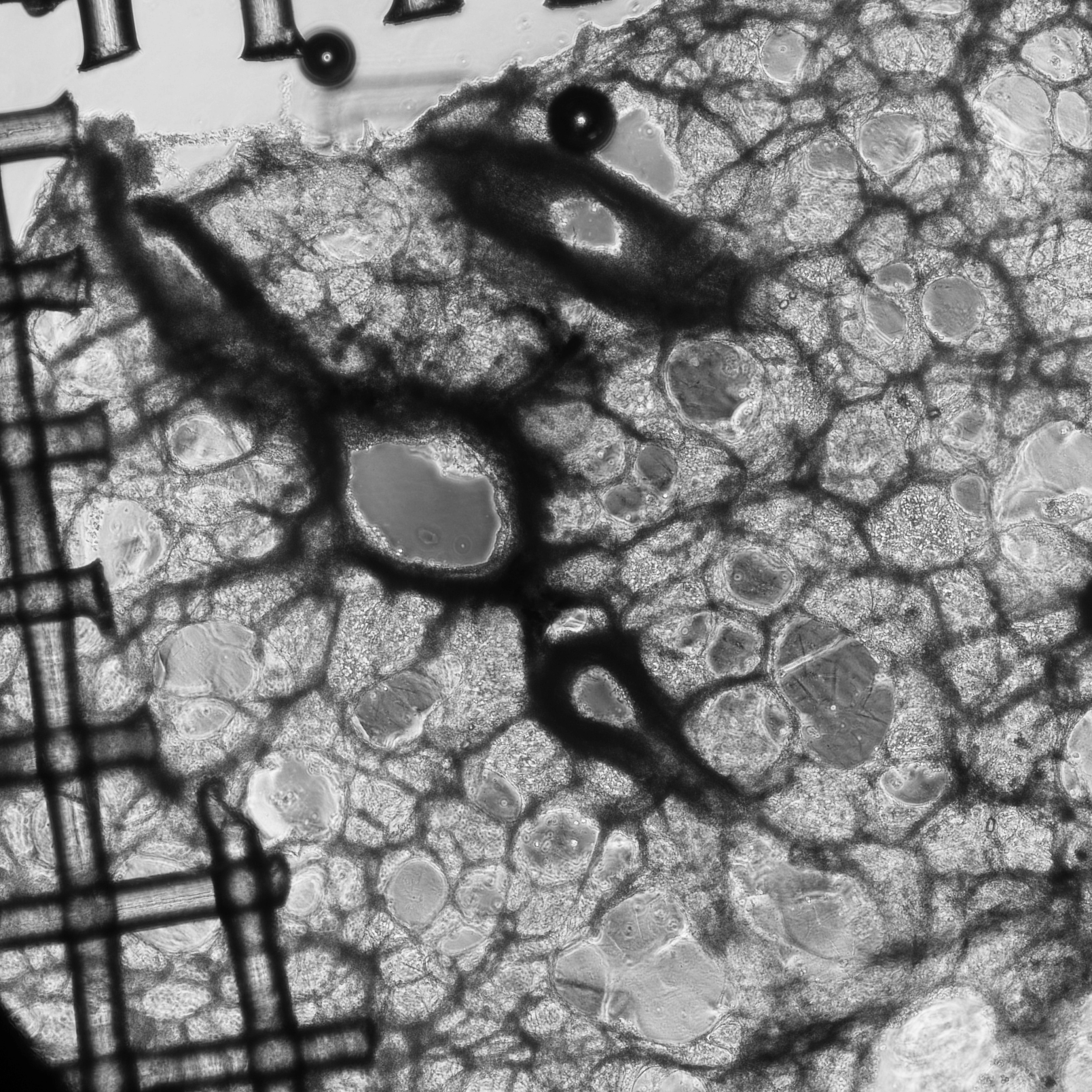

Supplement: Supplementary file 6 — Source data Fig. 5 [file 44321_2024_96_MOESM6_ESM.zip › Figure 5/5D/vessel01-endo-DMSO control right.tif]

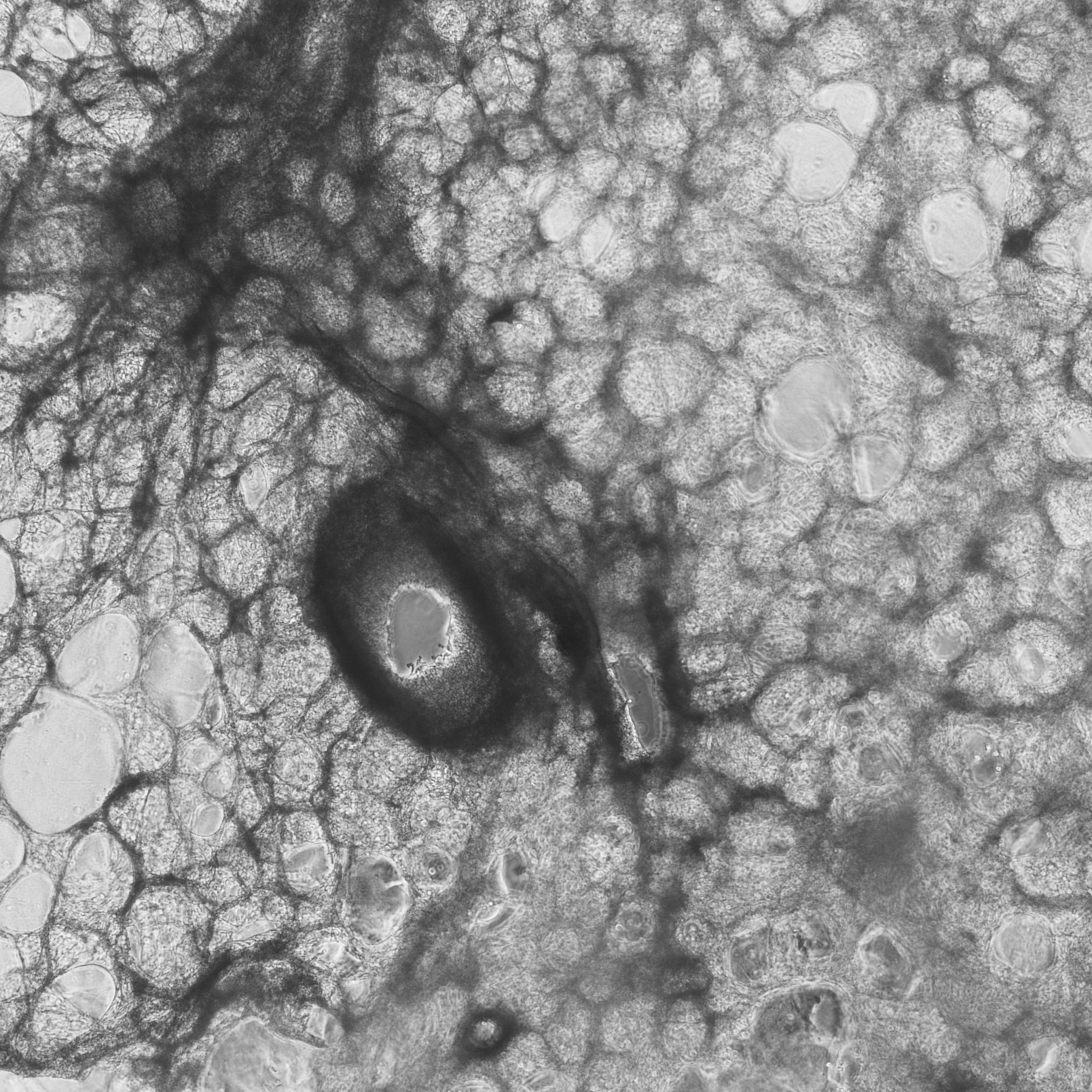

Supplement: Supplementary file 6 — Source data Fig. 5 [file 44321_2024_96_MOESM6_ESM.zip › Figure 5/5E/vessel07-baseline left.tif]

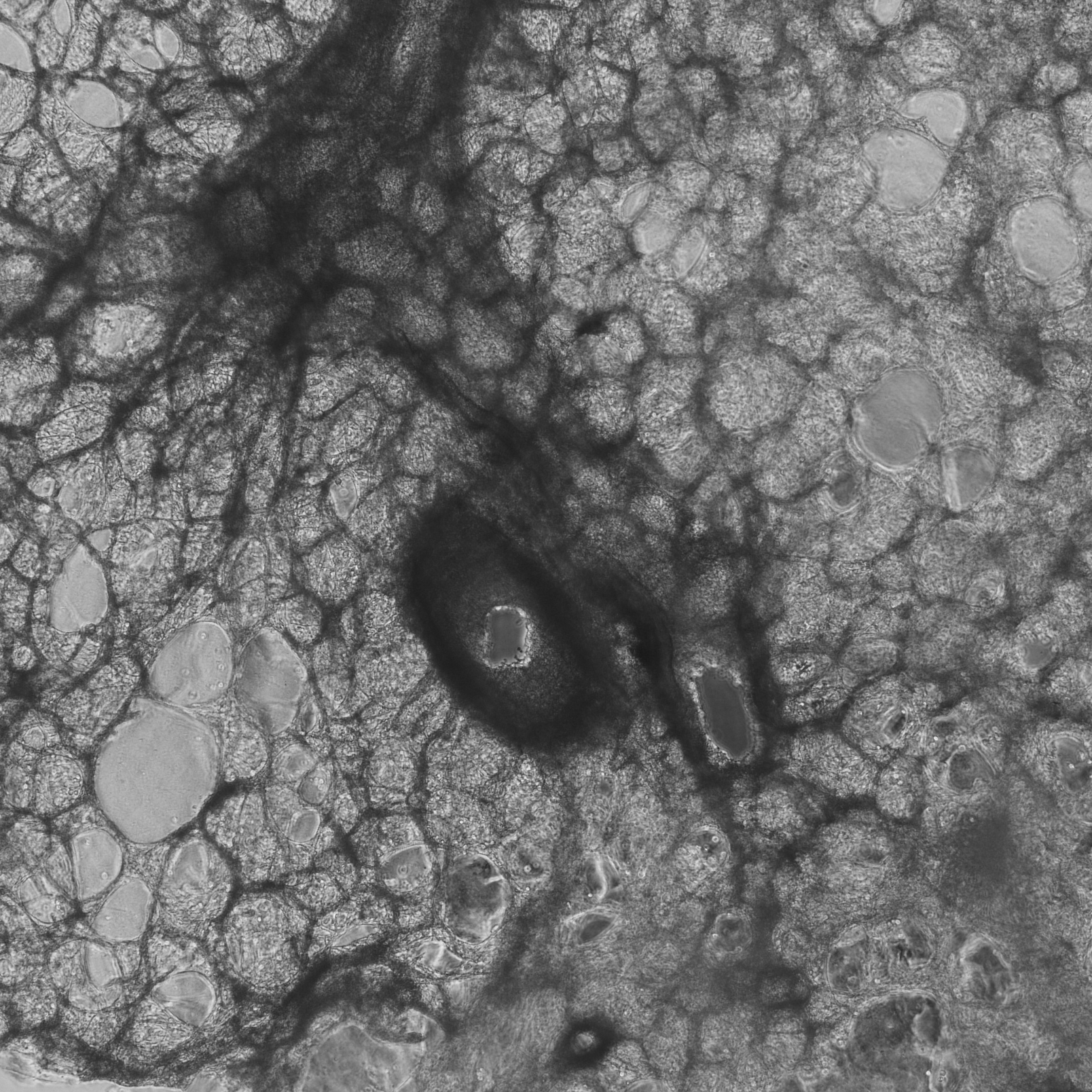

Supplement: Supplementary file 6 — Source data Fig. 5 [file 44321_2024_96_MOESM6_ESM.zip › Figure 5/5E/vessel07-endo middle.tif]

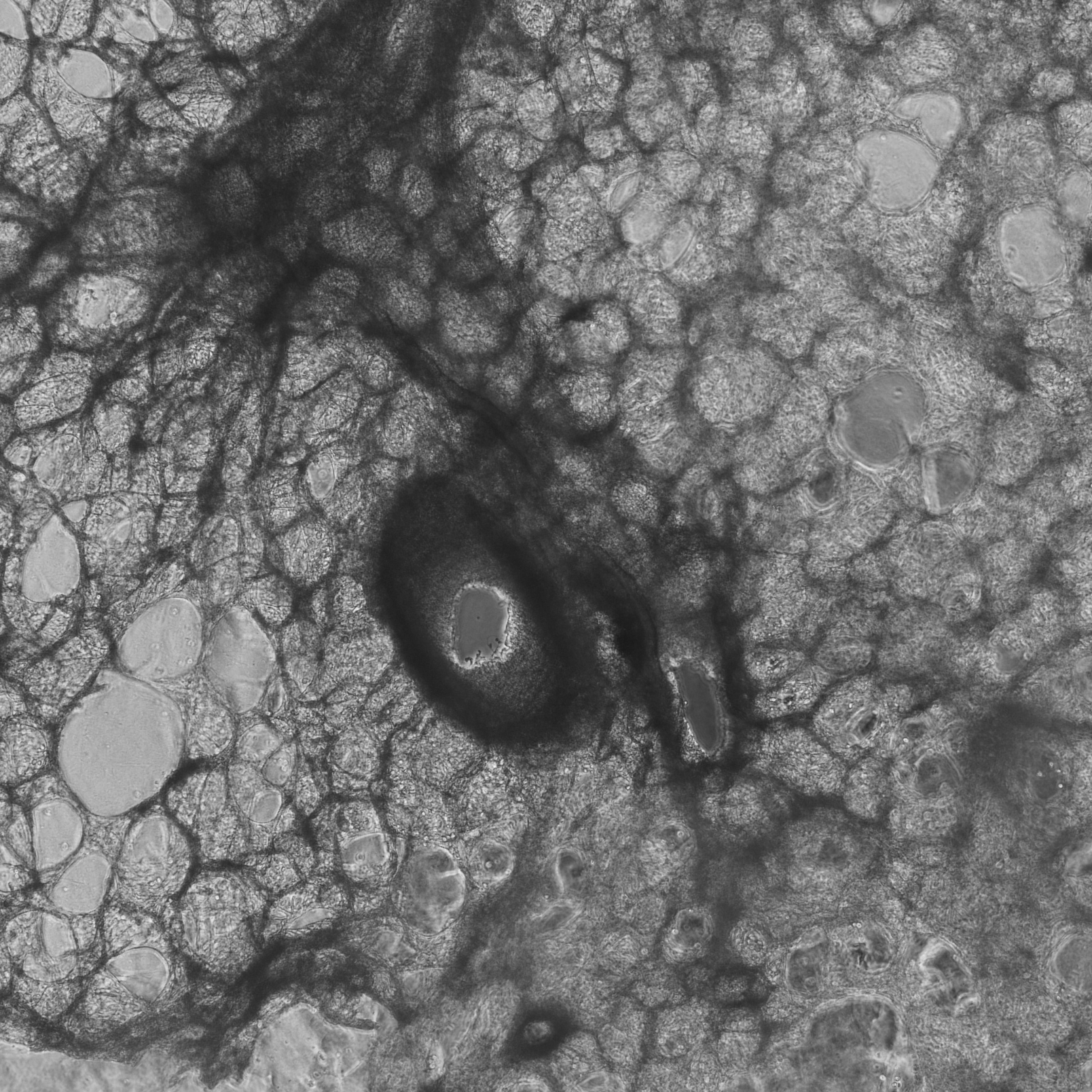

Supplement: Supplementary file 6 — Source data Fig. 5 [file 44321_2024_96_MOESM6_ESM.zip › Figure 5/5E/vessel07-endo-FR right.tif]

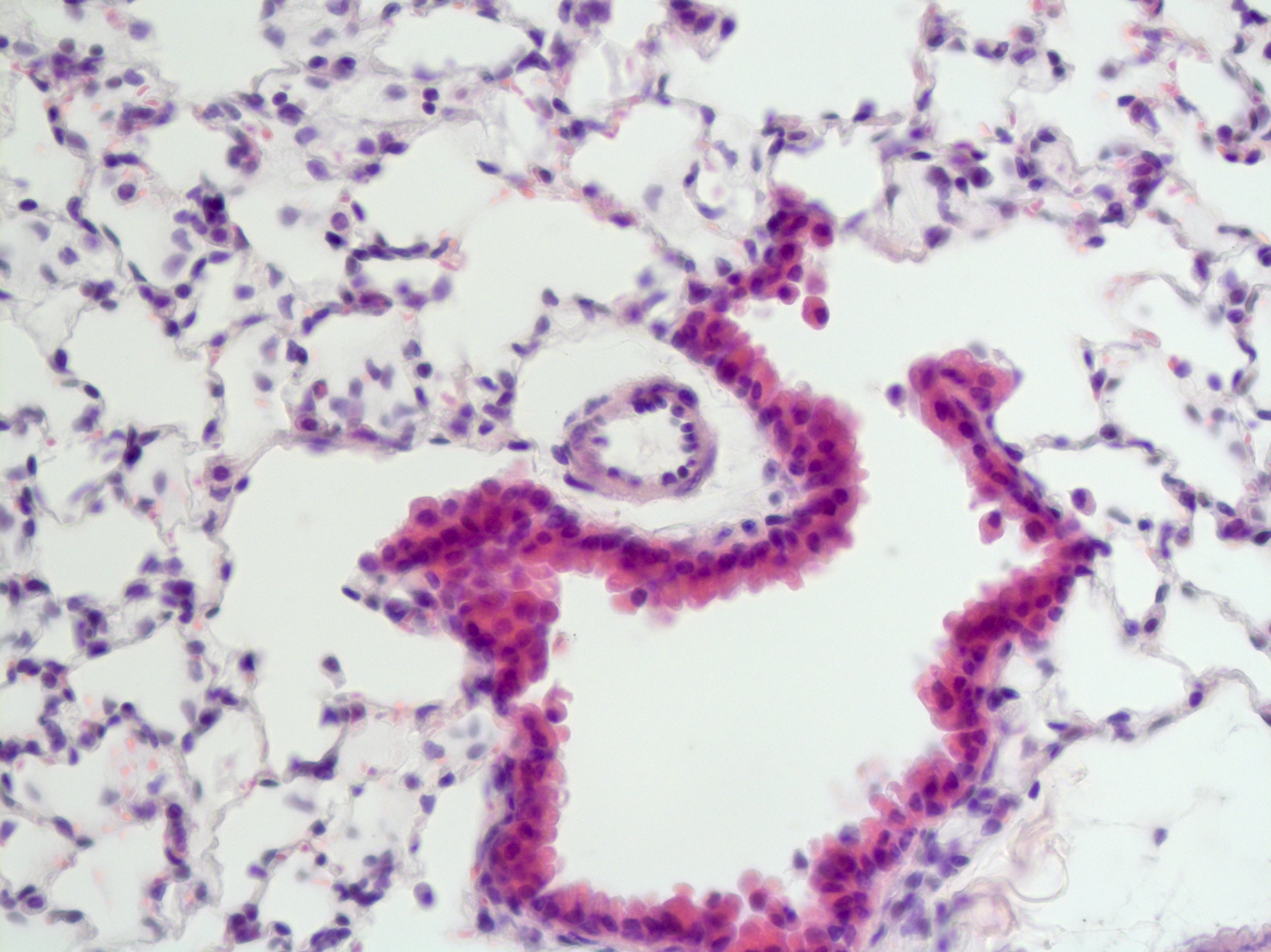

Supplement: Supplementary file 7 — Source data Fig. 6 [file 44321_2024_96_MOESM7_ESM.zip › Figure 6/6C/DMSO.tif]

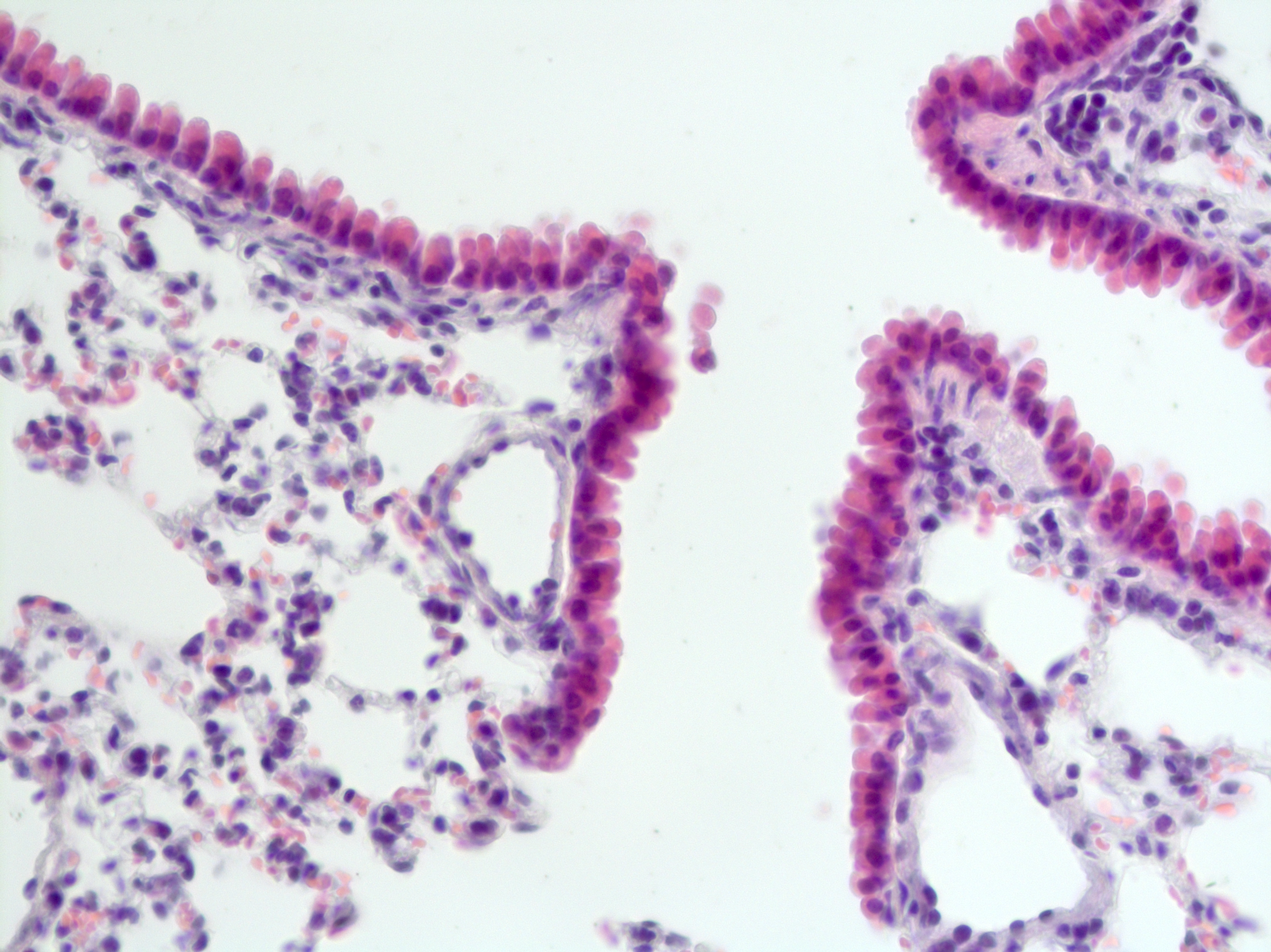

Supplement: Supplementary file 7 — Source data Fig. 6 [file 44321_2024_96_MOESM7_ESM.zip › Figure 6/6D/FR.tif]

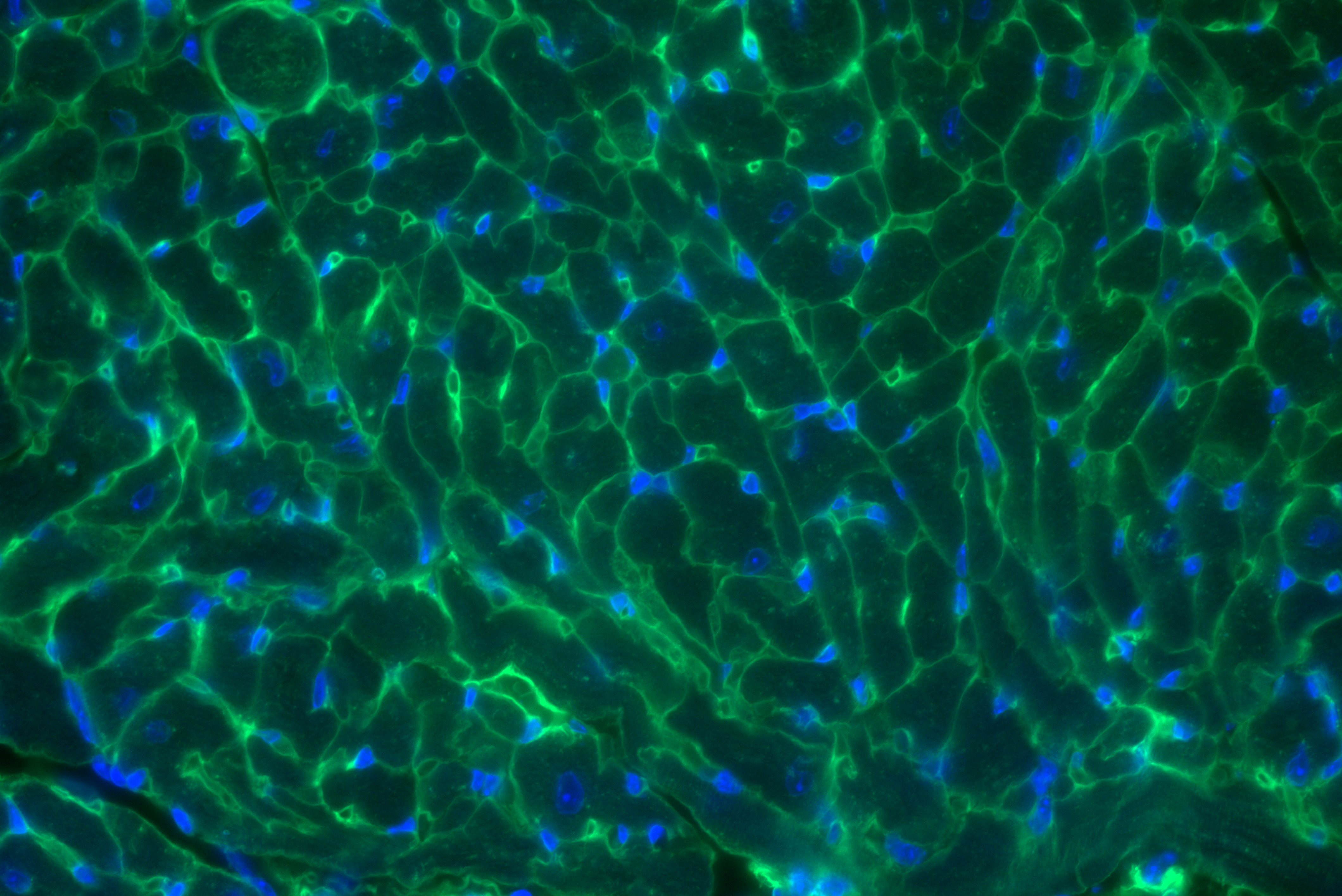

Supplement: Supplementary file 7 — Source data Fig. 6 [file 44321_2024_96_MOESM7_ESM.zip › Figure 6/6G/DMSO.tif]

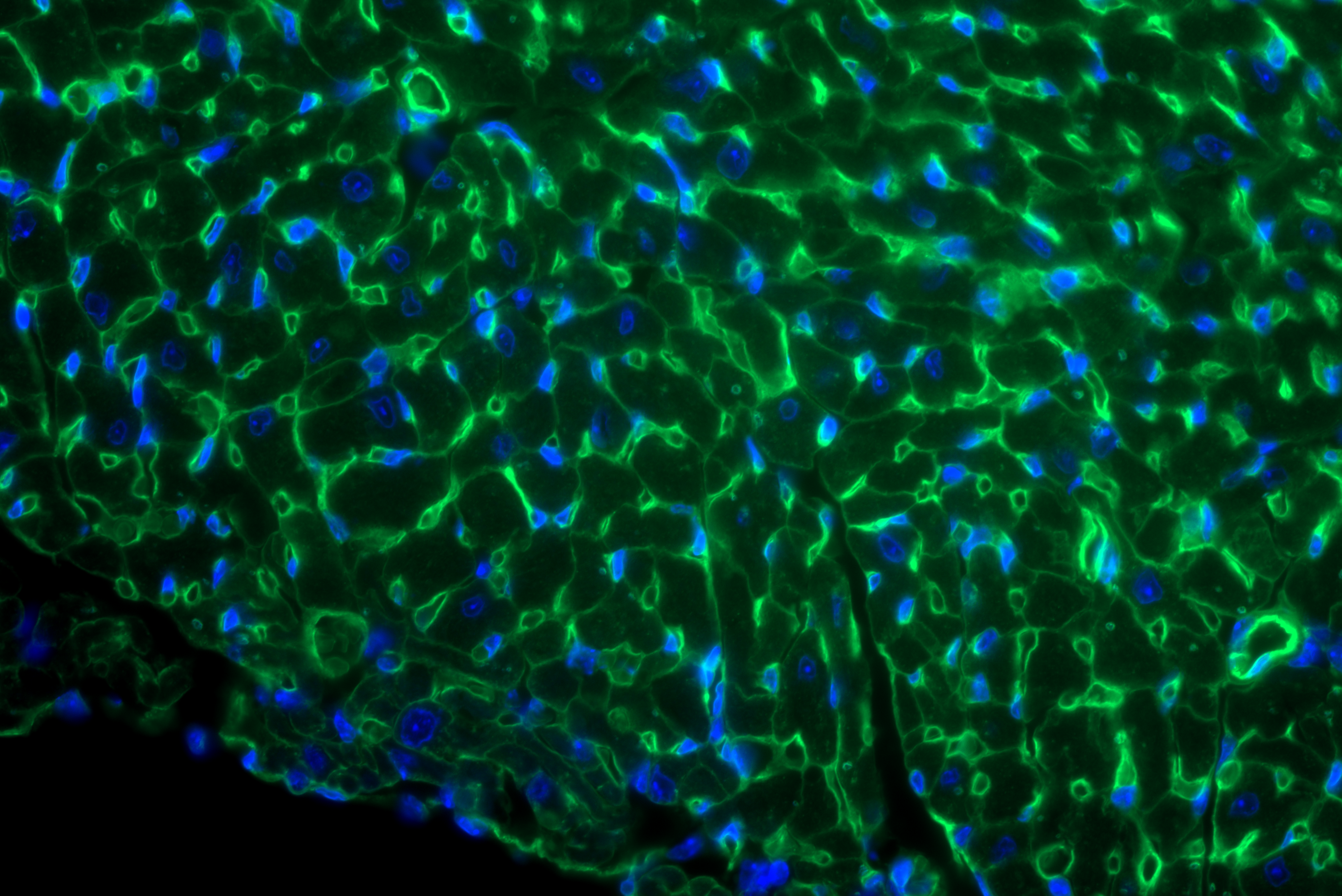

Supplement: Supplementary file 7 — Source data Fig. 6 [file 44321_2024_96_MOESM7_ESM.zip › Figure 6/6H/FR.tif]

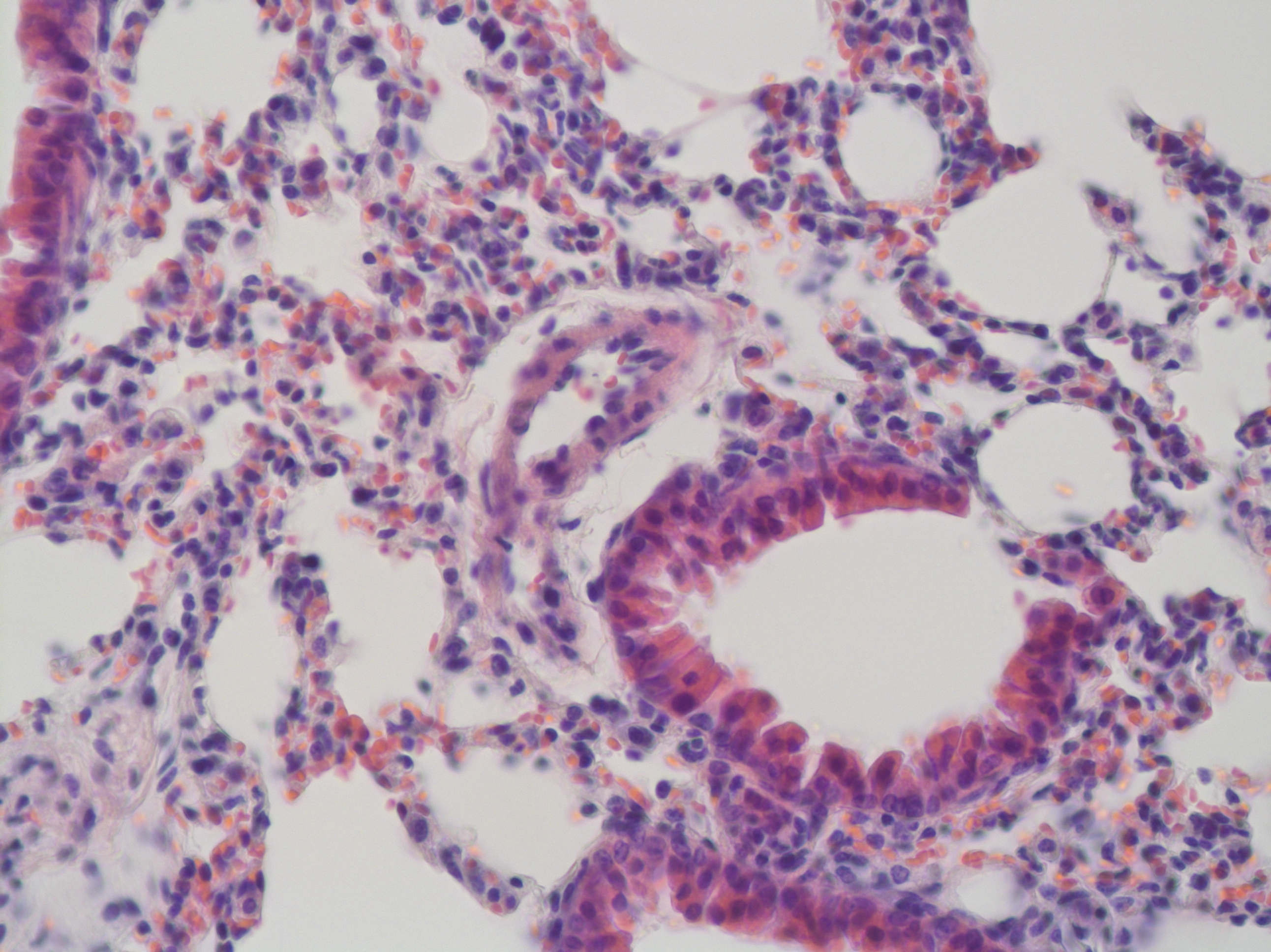

Supplement: Supplementary file 9 — Source data Fig. 8 [file 44321_2024_96_MOESM9_ESM.zip › Figure 8/8C/DMSO.tif]

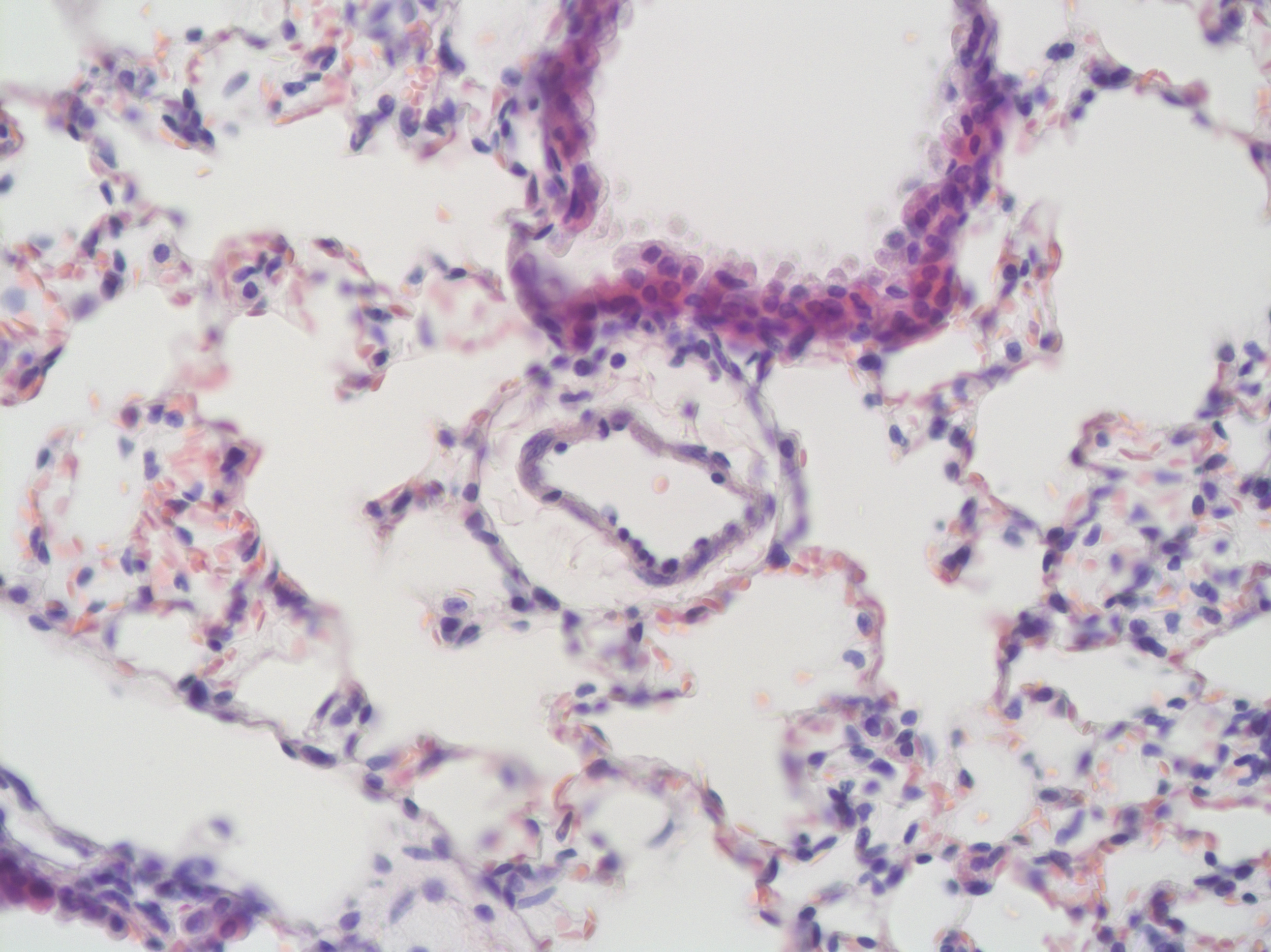

Supplement: Supplementary file 9 — Source data Fig. 8 [file 44321_2024_96_MOESM9_ESM.zip › Figure 8/8D/FR.tif]

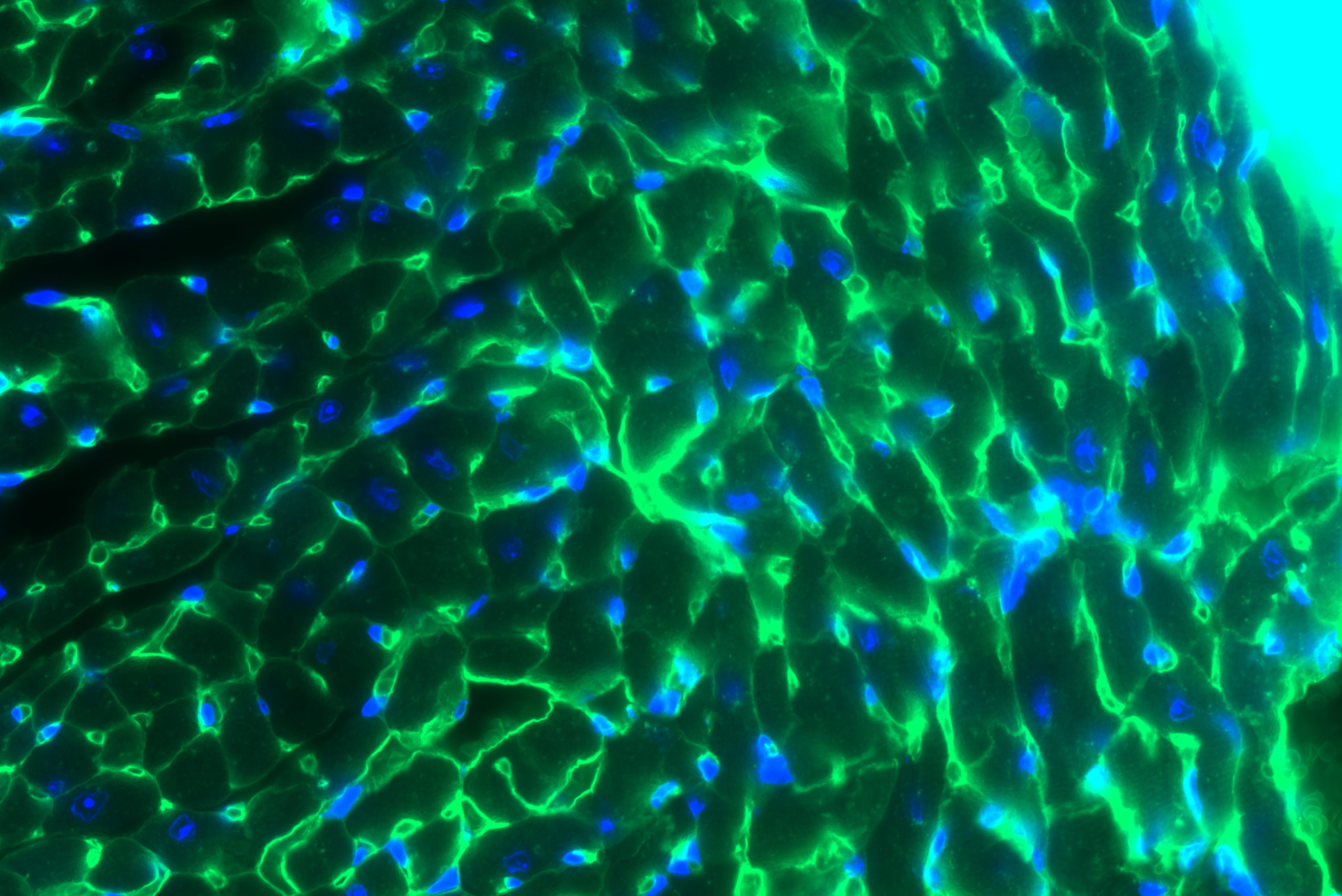

Supplement: Supplementary file 9 — Source data Fig. 8 [file 44321_2024_96_MOESM9_ESM.zip › Figure 8/8J/DMSO.tif]

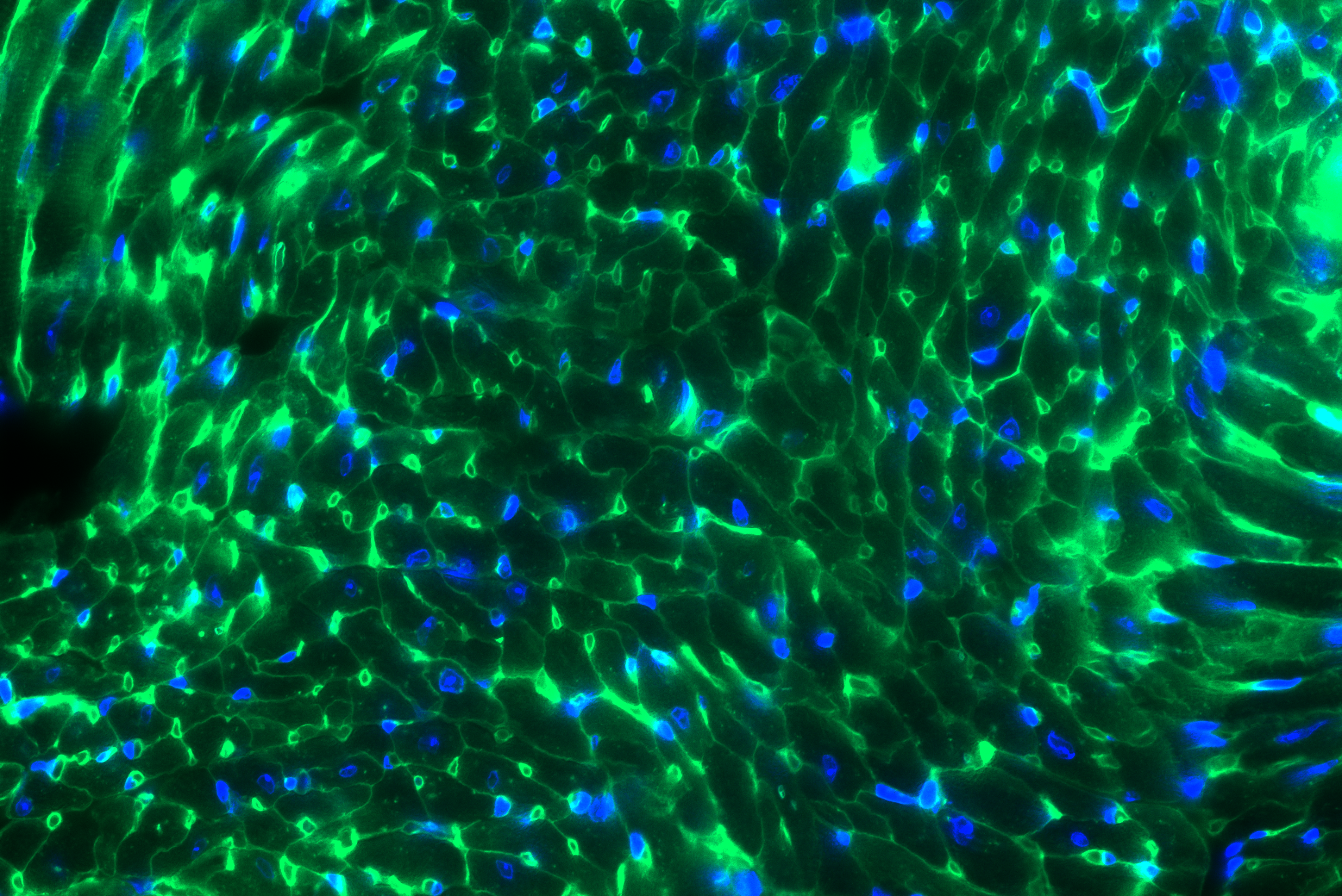

Supplement: Supplementary file 9 — Source data Fig. 8 [file 44321_2024_96_MOESM9_ESM.zip › Figure 8/8K/FR.tif]

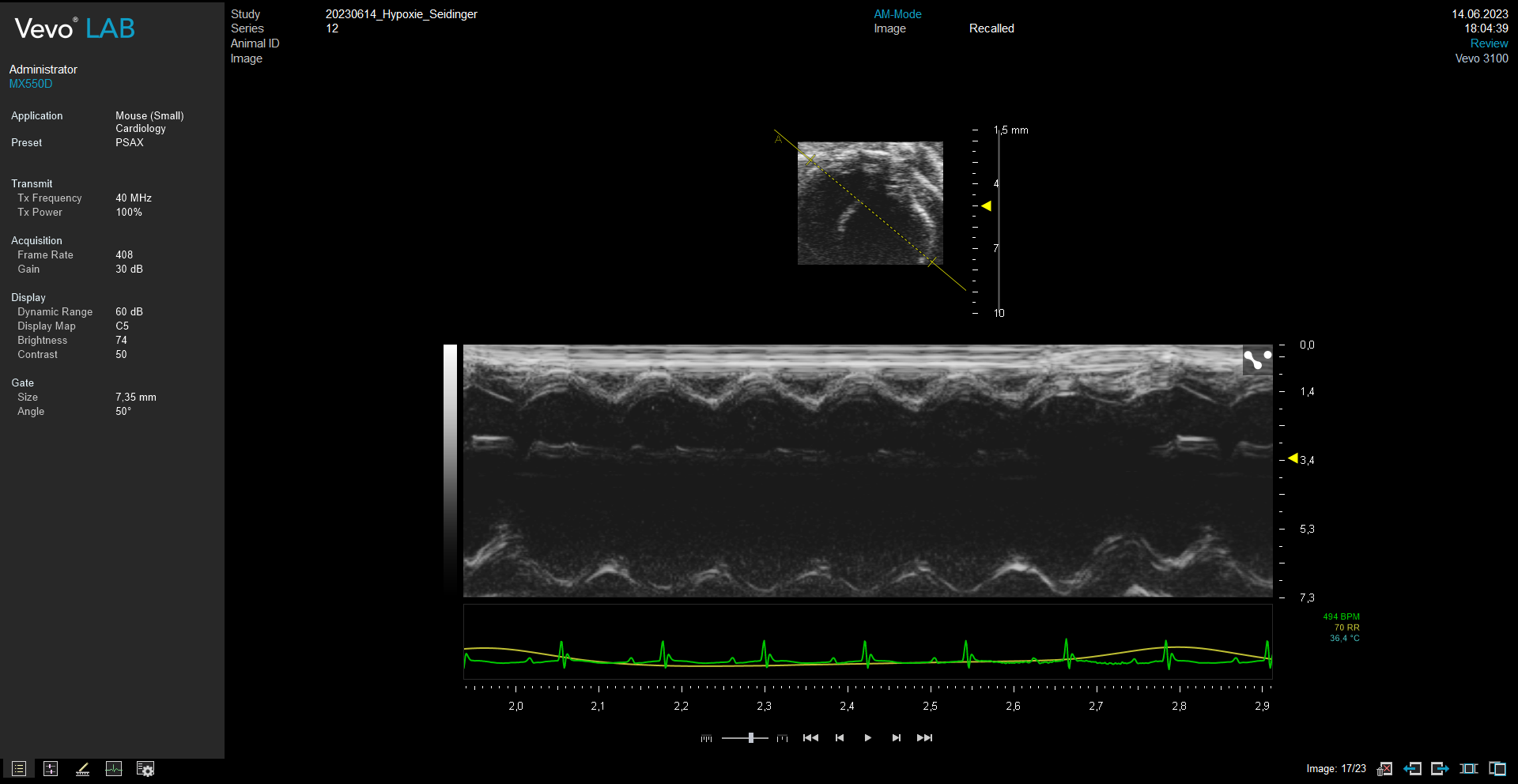

Supplement: Supplementary file 9 — Source data Fig. 8 [file 44321_2024_96_MOESM9_ESM.zip › Figure 8/8M/DMSO.tif]

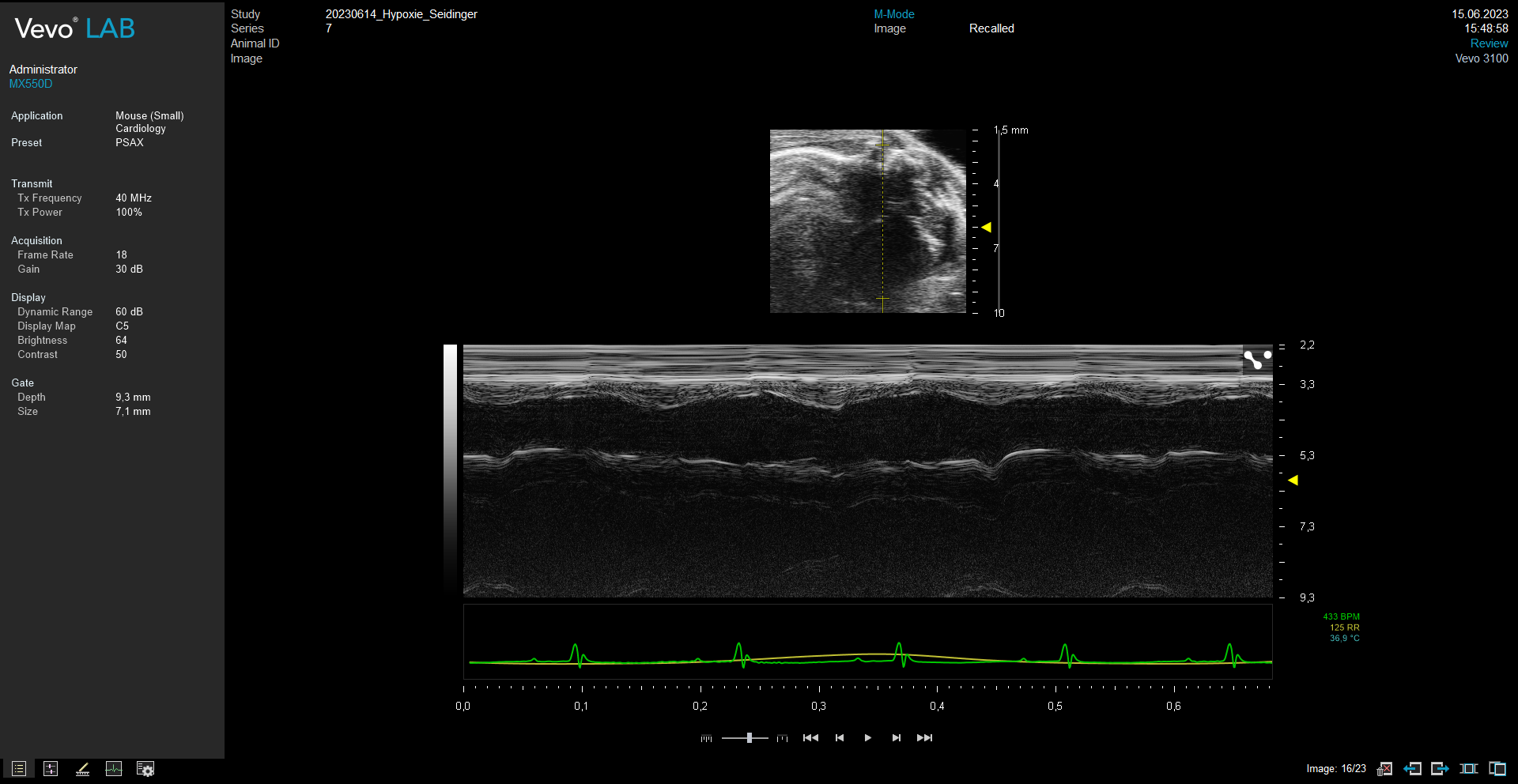

Supplement: Supplementary file 9 — Source data Fig. 8 [file 44321_2024_96_MOESM9_ESM.zip › Figure 8/8N/FR.tif]

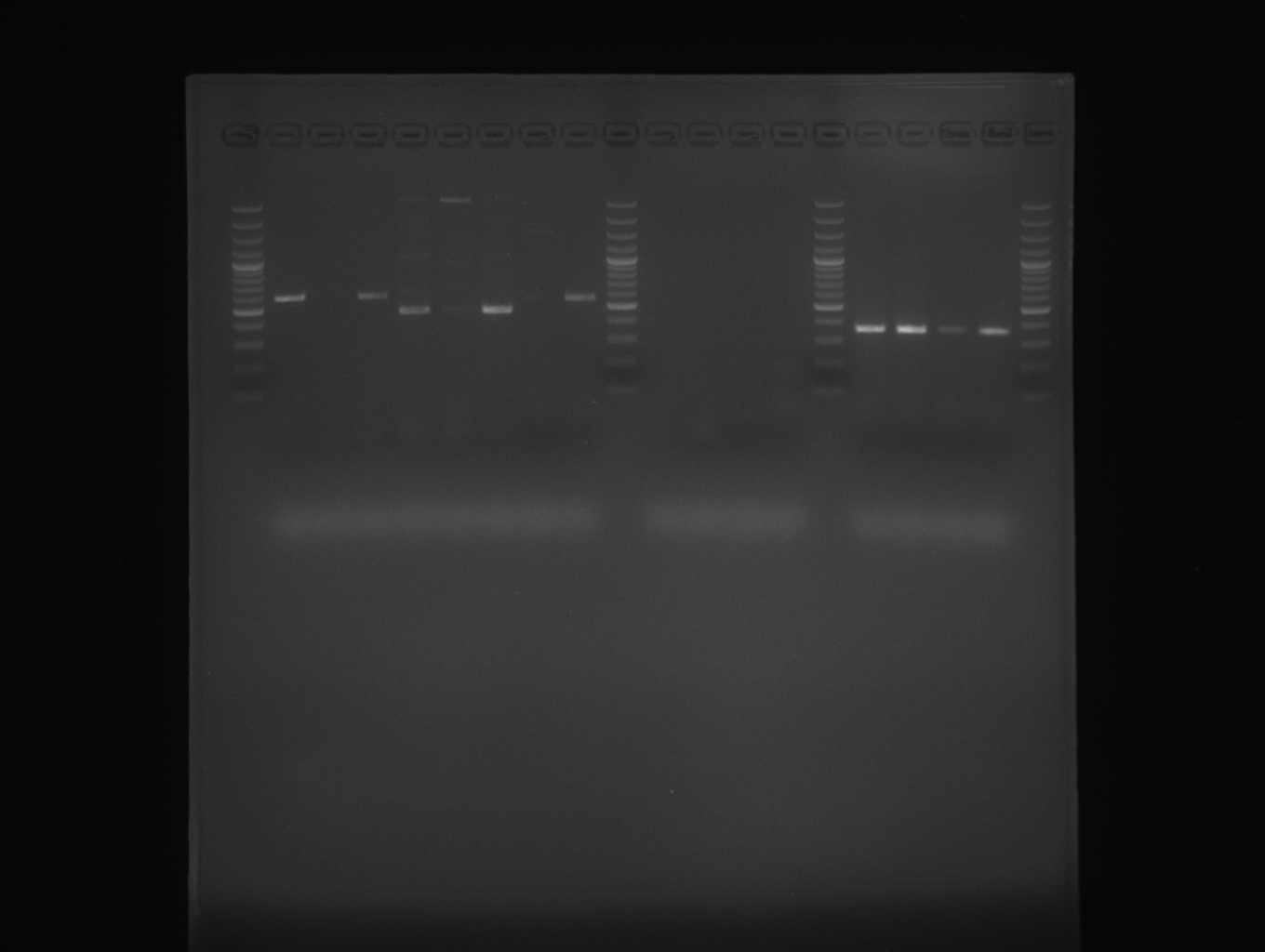

Supplement: Supplementary file 10 — EV Figure Source Data [file 44321_2024_96_MOESM10_ESM.zip › EMM-2023-18862 Expanded view figures/Figure EV1/EV1B/replicate/EV1B replicate.Tif]

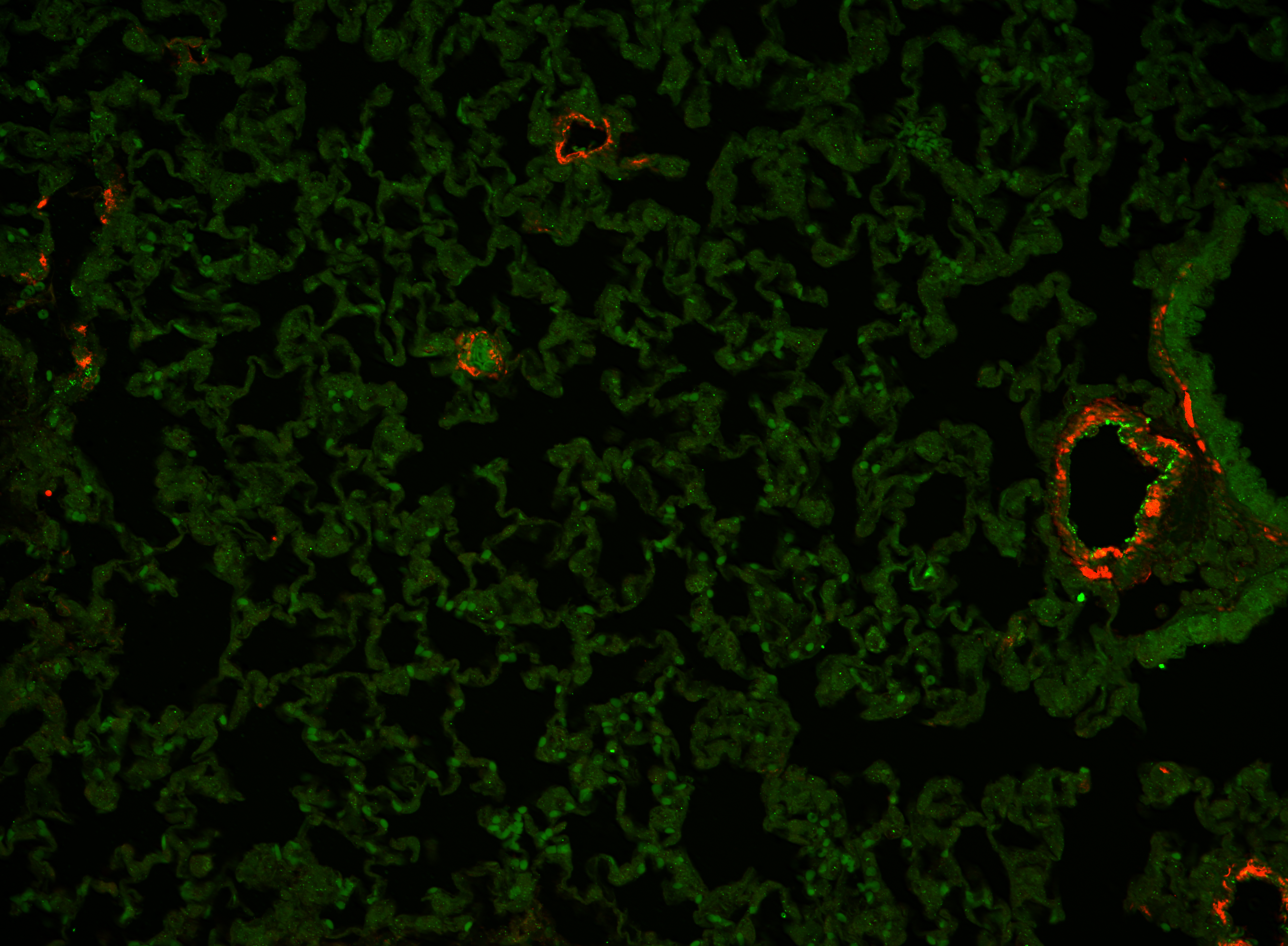

Supplement: Supplementary file 10 — EV Figure Source Data [file 44321_2024_96_MOESM10_ESM.zip › EMM-2023-18862 Expanded view figures/Figure EV5/EV5F/DMSO.tif]

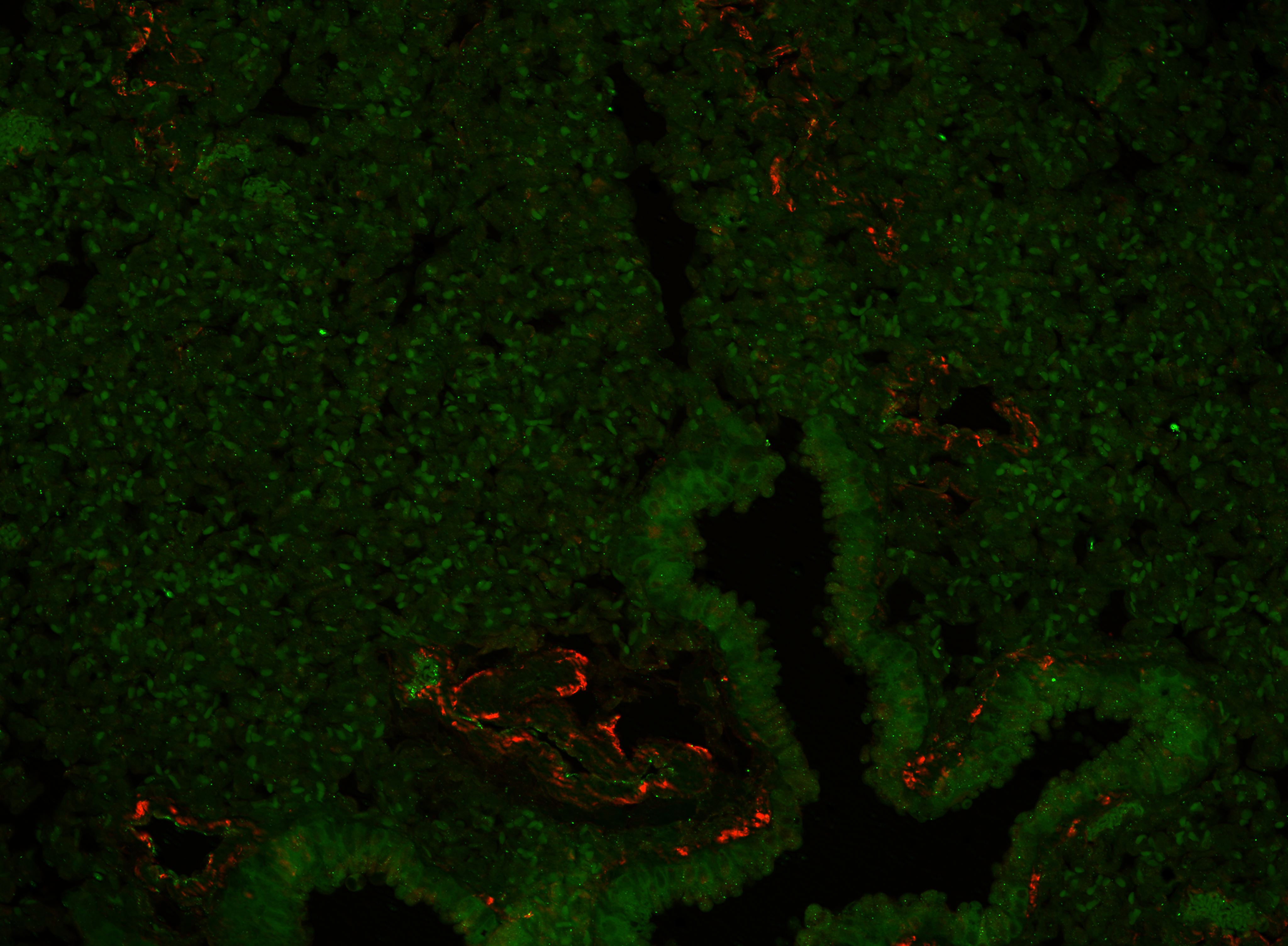

Supplement: Supplementary file 10 — EV Figure Source Data [file 44321_2024_96_MOESM10_ESM.zip › EMM-2023-18862 Expanded view figures/Figure EV5/EV5G/FR.tif]
