# Supplementary material for: Pharmacological Gq inhibition induces strong pulmonary vasorelaxation and reverses pulmonary hypertension
Source: EMBO Mol Med. 2024 Jul 8;16(8):7. doi: 10.1038/s44321-024-00096-0 (PMC11319782; doi:10.1038/s44321-024-00096-0)
Supplement: Supplementary file 11 — Expanded View Figures [file 44321_2024_96_MOESM11_ESM.pdf]

Expanded View Figures

Figure EV1. FR prevents 5-HT-induced constriction and $G\alpha_q/G\alpha_{11}$ can be downregulated by lentiviral transduction.

(**A**) Original traces of 5-HT dose–response curves (10^{-9} M–10^{-5} M) after pre-incubation with DMSO, Ket (10^{-6} M) or FR (10^{-6} M) in PAs. (**B**) PCR analysis of Gq protein subtypes in native mPASMCs and mPASMCs transduced with lentiviral sh-G11, sh-Gq RNA (negative controls). Murine lung tissue was used as positive control. (**C–F**) Statistical analysis of relative $G\alpha_{11}$ (**C**), $G\alpha_q$ (**D**), $G\alpha_i$ (**E**), and $G\alpha_s$ (**F**) mRNA expression in native mPASMCs ($n = 3$) and mPASMCs transduced with lentivirus (sh-control (ctrl), sh-G11, sh-Gq RNA or both, $n = 3$ independent experiments normalized to 18S housekeeping gene. (**G**, **H**) Original Western Blot (**G**) and analysis (**H**) of $G\alpha_{q/11/14}$ protein expression of native mPASMCs and mPASMCs transduced with lentivirus (sh-control (ctrl), sh-G11, sh-Gq RNA or both, $n = 3$ independent experiments). GAPDH was used as housekeeper. Data information: Values are expressed as mean ± SEM. (**C–F**, **H**) One-way ANOVA, Tukey's post hoc test. Source data are available online for this figure.

© The Author(s)

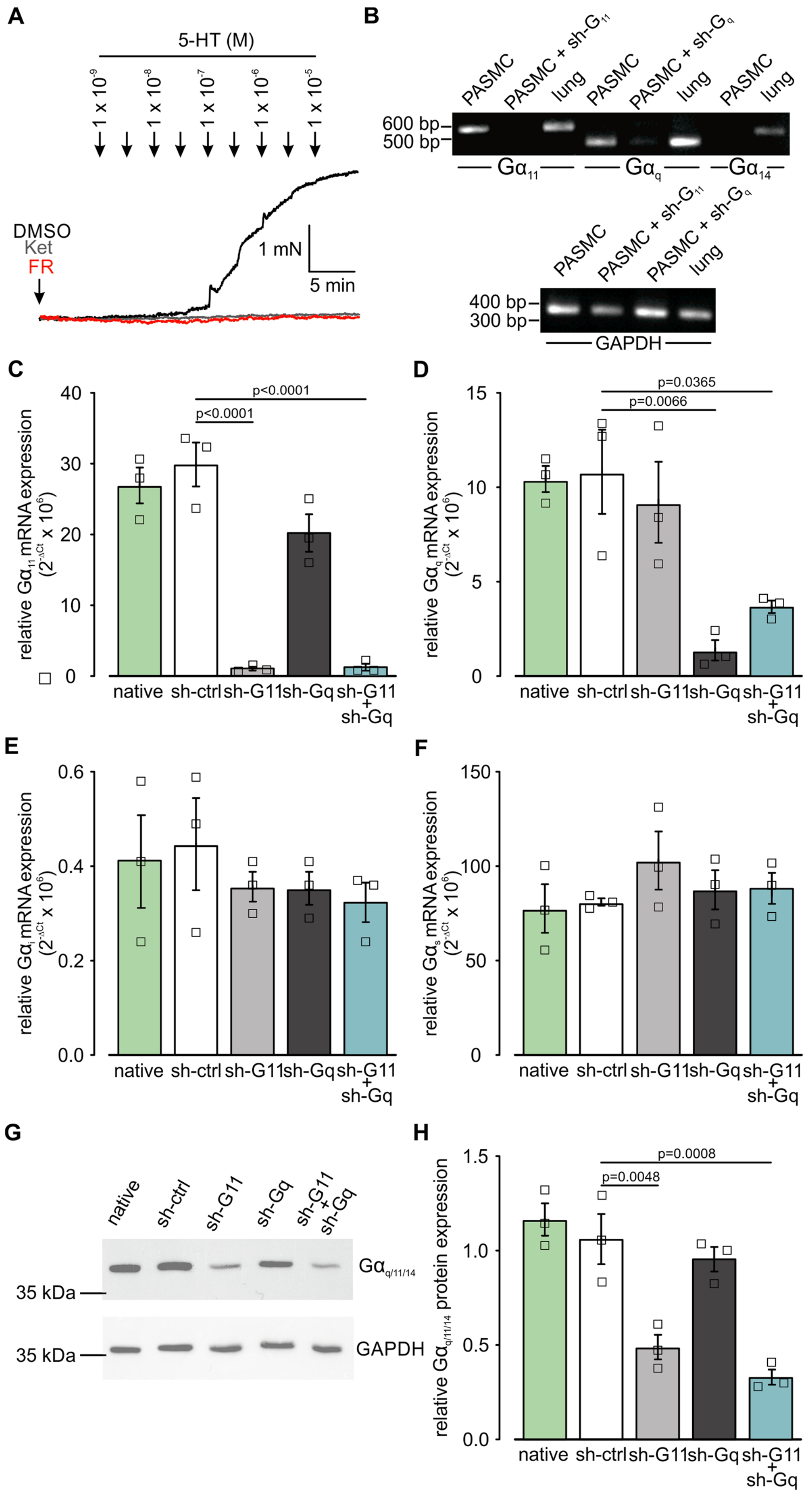
A
5-HT (M)
1 x 10-9
1 x 10-8
1 x 10-7
1 x 10-6
1 x 10-5
DMSO
Ket
FR
1 mN
5 min
B
PASMC
PASMC + sh-G11
lung
PASMC
PASMC + sh-Gq
lung
PASMC
lung
600 bp
500 bp
Gα11
Gαq
Gα14
PASMC
PASMC + sh-G11
PASMC + sh-Gq
lung
400 bp
300 bp
GAPDH
C
relative Gα11 mRNA expression (2-ΔCt x 10^6)
p<0.0001
p<0.0001
native
sh-ctrl
sh-G11
sh-Gq
sh-G11 + sh-Gq
D
relative Gαq mRNA expression (2-ΔCt x 10^6)
p=0.0365
p=0.0066
E
relative Gαi mRNA expression (2-ΔCt x 10^6)
F
relative Gαs mRNA expression (2-ΔCt x 10^6)
G
native
sh-ctrl
sh-G11
sh-Gq
sh-G11 + sh-Gq
Gαq/11/14
35 kDa
GAPDH
35 kDa
H
relative Gαq/11/14 protein expression
p=0.0008
p=0.0048

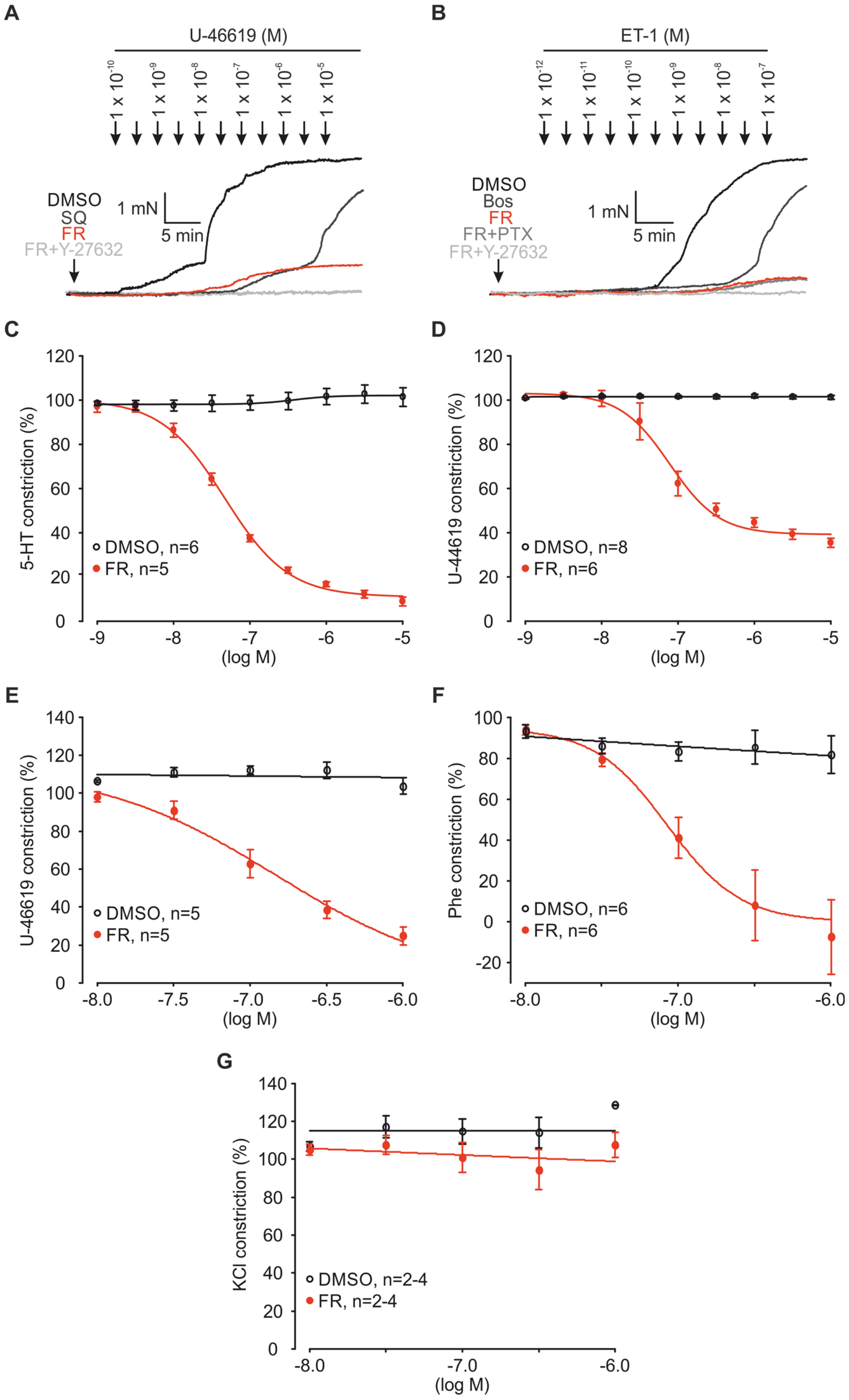
A
U-46619 (M)
1 x 10-10
1 x 10-9
1 x 10-8
1 x 10-7
1 x 10-6
1 x 10-5
DMSO
SQ
FR
FR+Y-27632
1 mN
5 min
B
ET-1 (M)
1 x 10-12
1 x 10-11
1 x 10-10
1 x 10-9
1 x 10-8
1 x 10-7
DMSO
Bos
FR
FR+PTX
FR+Y-27632
1 mN
5 min
C
5-HT constriction (%)
DMSO, n=6
FR, n=5
(log M)
D
U-44619 constriction (%)
DMSO, n=8
FR, n=6
(log M)
E
U-46619 constriction (%)
DMSO, n=5
FR, n=5
(log M)
F
Phe constriction (%)
DMSO, n=6
FR, n=6
(log M)
G
KCl constriction (%)
DMSO, n=2-4
FR, n=2-4
(log M)

Figure EV2. FR prevents and reverses Gq-mediated constriction in mouse and pig PAs.

(**A**) Original traces of U-46619 dose–response curves (10^{-10} M–10^{-5} M) after pre-incubation with DMSO, SQ (10^{-6} M), FR (10^{-6} M), or FR + Y-27632 (10^{-5} M) in mouse PAs. (**B**) Original traces of ET-1 dose–response curves (10^{-12} M–10^{-7} M) after pre-incubation with DMSO, Bos (10^{-6} M), FR (10^{-6} M), FR + PTX (1 μg/ml) or FR + Y-27632 (10^{-5} M) in mouse PAs. (**C**, **D**) Dose–response curves of DMSO and FR (10^{-9} M–10^{-5} M) after 5-HT (5×10^{-7} M, DMSO: $n = 6$, FR: $n = 5$, **C**) or U-46619 (10^{-7} M, DMSO: $n = 8$, FR: $n = 6$, **D**) pre-constriction in murine PAs. (**E–G**) Dose–response curves of DMSO and FR (10^{-8} M–10^{-6} M) after U-46619 (3×10^{-7} M, DMSO: $n = 5$, FR: $n = 5$, **E**), Phe (3×10^{-5} M, DMSO: $n = 6$, FR: $n = 6$, **F**), or KCl (3×10^{-2} M, DMSO: $n = 2$–4, FR: $n = 2$–4, **G**) pre-constriction in porcine PAs. Data information: Values are expressed as mean ± SEM. Source data are available online for this figure.

 © The Author(s)

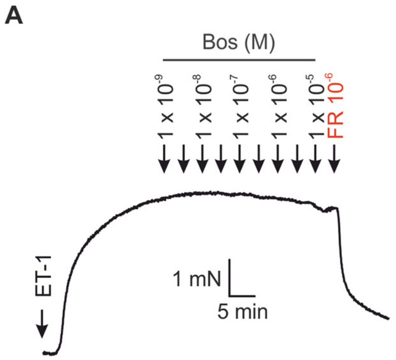

Figure EV3. FR strongly relaxes PAs ex vivo.

(**A**, **B**) Original traces of Bos (**A**) or Ilo (**B**) dose–response curves (10^{-9} M – 10^{-5} M) followed by single dose FR (10^{-6} M) application after pre-constriction with ET-1 (3×10^{-9} M) in mouse PAs.

© The Author(s)

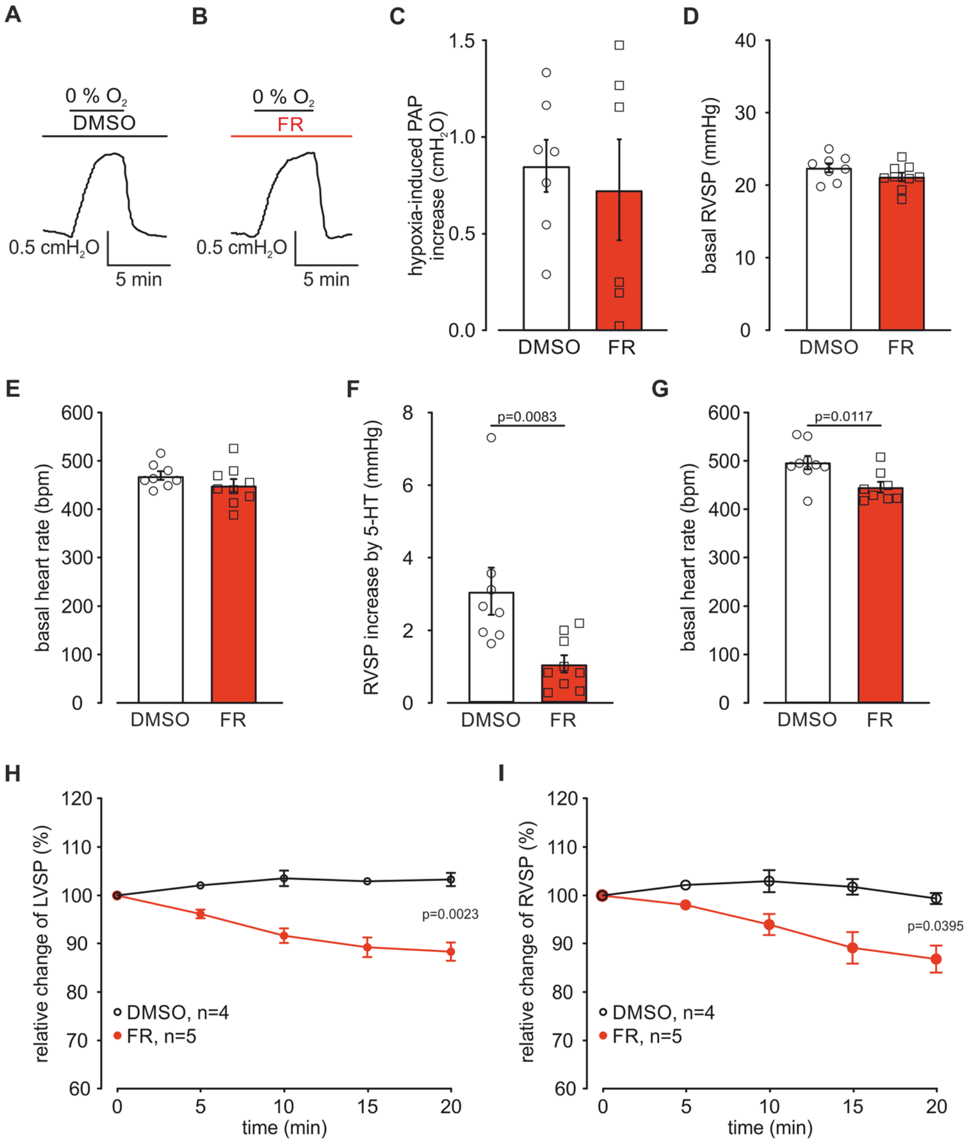

Figure EV4. FR does not affect HPV but reduces RVSP in vivo.

(**A**, **B**) Original traces of PAP in the IPL model during perfusion with DMSO (**A**) or FR (10^{-6} M, **B**) and exposure to hypoxic air (0% O_2/100% N_2). (**C**) Statistical analysis of PAP increase evoked by hypoxic air during DMSO ($n = 7$ mice) or FR ($n = 6$ mice) perfusion. (**D**) Statistical analysis of basal RVSP 1 h after DMSO ($n = 8$) or FR (2.5 µg/mouse, $n = 8$) i.t. application in healthy mice housed under normoxic (21% O_2) conditions. (**E**) Statistical analysis of basal heart rate in these mice (DMSO: $n = 8$; FR: $n = 8$). (**F**) Statistical analysis of RVSP increase in response to 5-HT (5×10^{-3} M, 10 µl) i.v. bolus injection in these mice (DMSO: $n = 8$; FR: $n = 8$). (**G**) Basal heart rate 1 h after DMSO ($n = 9$) or FR (2.5 µg/mouse, 1 h before, $n = 8$) application in mice with pre-existing Hx-induced PH (DMSO: $n = 9$; FR: $n = 8$). (**H**, **I**) Relative change of LVSP (**H**) and RVSP (**I**) after acute DMSO ($n = 4$) or FR (10 µg/mouse i.p., $n = 5$) application in mice with pre-existing Hx-induced PH. Data information: Values are expressed as mean ± SEM. (**C–G**) Unpaired student's t-test. (**H**, **I**) Two-way ANOVA, Bonferroni post hoc test. Source data are available online for this figure.

 © The Author(s)

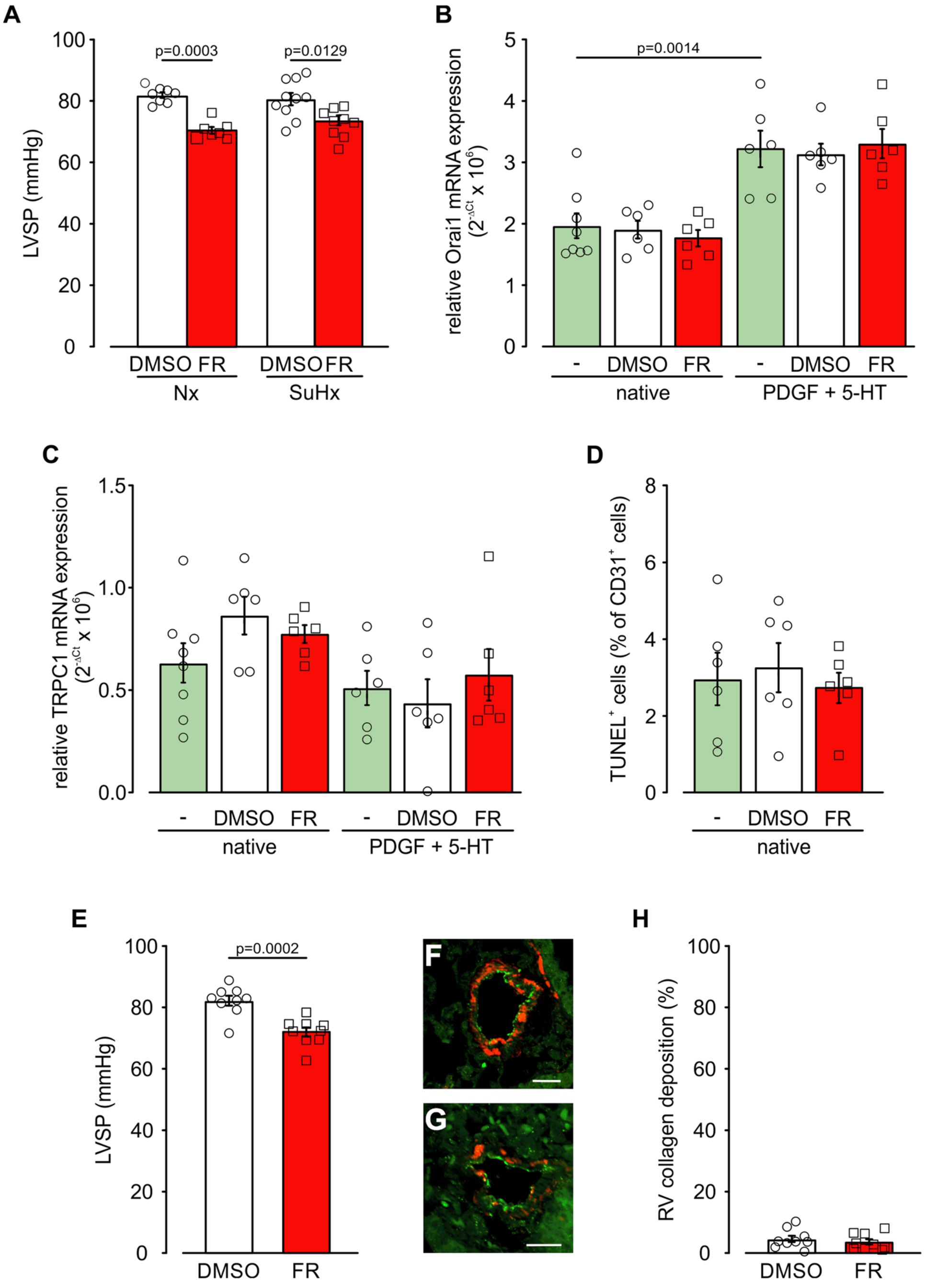

Figure EV5. FR effects on Hx-induced PH in vivo and mPASMCs as well as mLECs in vitro.

(**A**) Statistical analysis of LVSP in mice treated with the solvent DMSO or FR (10 μg/mouse i.p., Monday to Friday) during exposure to Nx (21% O_2, DMSO: $n = 8$, FR: $n = 7$) or SuHx (10% O_2, DMSO: $n = 10$, FR: $n = 10$) for 3 weeks. (**B**, **C**) Statistical analysis of relative Orai1 (**B**) and TRPC1 (**C**) mRNA expression in native mPASMCs ($n = 8$) and mPASMCs treated with solvent DMSO or FR (10^{-6} M) with or without additional PDGF (40 ng/ml) + 5-HT (10^{-6} M) stimulation for 12 h, each $n = 6$ normalized to 18 S housekeeping gene, ns indicate different wells derived from at least two different passages. (**D**) Amount of TUNEL$^+$ CD31$^+$ mLECs after 2 days without treatment ($n = 6$) or with DMSO ($n = 6$) or FR (10^{-6} M, $n = 6$) treatment. (**E**) Statistical analysis of LVSP in mice treated with the solvent DMSO ($n = 9$) or FR (10 μg/mouse i.p., Monday to Friday, $n = 9$) in the last 2 weeks of 5 weeks SuHx exposure. (**F**, **G**) vWF/α-SMAC staining of PAs in lung sections from SuHx-DMSO (**F**) and SuHx-FR-treated mice (**G**), scale bars: 20 μm. (**H**) Statistical analysis of collagen deposition in the right ventricle in mice treated with the solvent DMSO ($n = 9$) or FR (10 μg/mouse i.p., Monday to Friday, $n = 9$) in the last 2 weeks of 5 weeks SuHx exposure. Data information: Values are expressed as mean ± SEM. (**A–D**) One-way ANOVA, Tukey's post hoc test, (**E**, **H**) Unpaired student's t-test. Source data are available online for this figure.
